# Pharmacoinformatics-based investigation of bioactive compounds of *Rasam* (South Indian recipe) against human cancer

**DOI:** 10.1038/s41598-021-01008-9

**Published:** 2021-11-02

**Authors:** Arjun Kumar Kalimuthu, Theivendren Panneerselvam, Parasuraman Pavadai, Sureshbabu Ram Kumar Pandian, Krishnan Sundar, Sankaranarayanan Murugesan, Damodar Nayak Ammunje, Sattanathan Kumar, Sankarganesh Arunachalam, Selvaraj Kunjiappan

**Affiliations:** 1 Department of Biotechnology, Kalasalingam Academy of Research and Education, Krishnankoil, Tamil Nadu 626126 India; 2 Department of Pharmaceutical Chemistry, Swamy Vivekanandha College of Pharmacy, Elayampalayam, Tiruchengodu, Tamil Nadu 637205 India; 3 Department of Pharmaceutical Chemistry, Faculty of Pharmacy, M.S. Ramaiah University of Applied Sciences, M S R Nagar, Bengaluru, Karnataka 560054 India; 4 Department of Pharmacy, Birla Institute of Technology and Science Pilani, Pilani Campus, Vidya Vihar, Pilani, Rajasthan 333031 India; 5 Department of Pharmacology, Faculty of Pharmacy, M.S. Ramaiah University of Applied Sciences, M S R Nagar, Bengaluru, Karnataka 560054 India; 6 Deparment of Pharmaceutical Chemistry, Paavai College of Pharmacy and Research, Namakkal, Tamil Nadu 637018 India

**Keywords:** Cancer prevention, Computational biology and bioinformatics

## Abstract

Spice-rich recipes are referred to as “functional foods” because they include a variety of bioactive chemicals that have health-promoting properties, in addition to their nutritional value. Using pharmacoinformatics-based analysis, we explored the relevance of bioactive chemicals found in *Rasam* (a South Indian cuisine) against oxidative stress-induced human malignancies. The *Rasam* is composed of twelve main ingredients, each of which contains a variety of bioactive chemicals. Sixty-six bioactive compounds were found from these ingredients, and their structures were downloaded from Pubchem. To find the right target via graph theoretical analysis (mitogen-activated protein kinase 6 (MAPK6)) and decipher their signaling route, a network was built. Sixty-six bioactive compounds were used for in silico molecular docking study against MAPK6 and compared with known MAPK6 inhibitor drug (PD-173955). The top four compounds were chosen for further study based on their docking scores and binding energies. In silico analysis predicted ADMET and physicochemical properties of the selected compounds and were used to assess their drug-likeness. Molecular dynamics (MD) simulation modelling methodology was also used to analyse the effectiveness and safety profile of selected bioactive chemicals based on the docking score, as well as to assess the stability of the MAPK6-ligand complex. Surprisingly, the discovered docking scores against MAPK6 revealed that the selected bioactive chemicals exhibit varying binding ability ranges between − 3.5 and − 10.6 kcal mol^−1^. MD simulation validated the stability of four chemicals at the MAPK6 binding pockets, including Assafoetidinol A (ASA), Naringin (NAR), Rutin (RUT), and Tomatine (TOM). According to the results obtained, fifty of the sixty-six compounds showed higher binding energy (− 6.1 to − 10.6 kcal mol^−1^), and four of these compounds may be used as lead compounds to protect cells against oxidative stress-induced human malignancies.

## Introduction

Cancer is defined as unregulated cell or tissue growth that may spread to other parts of the body. It is the second greatest cause of mortality in the world, behind cardiovascular illnesses, and the number of cases continues to rise^1^. According to the GLOBOCON-2020 report, there are around 19.30 million new cancer cases diagnosed and 10.00 million cancer deaths worldwide^2^. A range of modifiable health behaviours, such as high fat and simple carbohydrate diet, irregular eating habits as well as poor physical activity contributes to the sudden rise in cancer incidences^3^. Several studies have shown that dysregulated nutrition and sedentary life style are key factors in the cellular redox process, resulting in unwanted by-products such as reactive oxygen species (ROS), reactive nitrogen species (RNS), and DNA reactive aldehyde^4–6^. In mitochondria, ROS is an unavoidable by-product of oxidative phosphorylation^7^. ROS is a two-edged sword that has both helpful (at low concentration) and harmful (at high concentration) properties. At low concentration, ROS regulates cellular activities such as cell cycle, proliferation, differentiation, migration, and death while an increased quantity of ROS may damage proteins, nucleic acids, lipids, membranes, and organelles, it also reduces cell viability and causes apoptosis^8, 9^.

The production of reactive oxygen species (ROS) in cells is neutralized by a number of antioxidant defense mechanisms. The detoxifying enzymes and antioxidant enzymes protect cells and tissues from toxins and oxidative stress^10^. Oxidative stress sensitive genes accomplish ROS scavenging by secreting antioxidant enzymes including superoxide dismutase (SOD), catalase, glutathione peroxidase, peroxiredoxins, and other non-enzymatic compounds such as flavonoids, carotenoids, glutathione, α-lipoic acid, iron chelators, vitamins A, C and E^11^. Furthermore, increased levels of intracellular ROS beyond a certain threshold cause down regulation of cellular antioxidant pathways and enzyme systems, resulting in malignant transformation via various molecular targets such as nuclear factor-B (NF-B), nuclear factor E2 (erythroid-derived 2)-related factor 2 (Nrf2), Kelch like-ECH-associated protein 1 (Keap1), mitogen-activated protein kinases (MAPKs) and phosphoinositide 3-kinase (PI3K)^12^.

The MAPKs are a class of serine/threonine protein kinases that play important roles in controlling extracellular signaling into a wide range of cellular processes^13^. Based on their structure and functions, they are classified into conventional and atypical MAPKs. Conventional MAPKs, such as, extracellular signal–regulated kinase (ERK) 1/2 and p38 isoforms (α, β, γ, and δ), and atypical MAPKs are ERK3/4 and ERK7/8^14^. The ERK 1/2 and p38 MAPK pathways have been targeted by numerous drugs to battle the various types of cancer with some clinical success. While compared with ERK 1/2 (conventional) MAPKs, much less work has been explored on ERK3, also known as MAPK6 signaling, and its cellular functions^15^. The ERK3 has significant physiological functions, including pulmonary differentiation, T cell activation, and angiogenesis^16^. In addition, MAPK6 has been connecting a series of signaling cascades and play a major role in the migration and invasiveness of certain types of cancers. MAPK6 is essential for production of several cellular factors including interleukin-8 (IL-8), in both, normal and tumorigenic cells^16^. MAPK6 is a widely expressed protein in all tissues with highest expression levels detected in skeletal muscle, brain, and gastrointestinal tract. MAPK6 interacts with and phosphorylates steroid receptor coactivator 3 (SRC-3), an oncogenic protein overexpressed in multiple human cancers at the amino acid residue, serine 857 (S857)^17^.

Spices are used in cuisine all around the globe for their taste, flavour and their health advantages^18^. Because, they contain numerous bioactive components, certain spices have been utilized in Indian traditional medicine to prevent and cure numerous ailments, including cancer^19^. Capsaicin (red pepper)^1^, curcumin (turmeric)^20^, piperine (black pepper)^21^, lycopene (tomato)^22^, myricetin (tea)^23^, and rutin (buckwheat)^24^ are a few examples of bioactive chemicals that have been shown to possess antioxidant and anticancer properties. “*Rasam*” is a famous South Indian spicy soup that has been made fresh every day and served with rice^25^. Tamarind, red pepper, black pepper, cumin seed, fenugreek, asafoetida, garlic, tomato, coriander, curry leaves, sesame oil, and mustard are the main flavors (spices) of *Rasam*^26^. These spices that used to make *Rasam*, a "functional food", include a plethora of bioactive chemicals that have been linked to improved tumor prognosis^27^. The synergistic activity of a mixed bioactive chemicals is always greater than that of a single component^28^. Furthermore, these bioactive chemicals function via many signaling pathways and display anticancer activity by blocking certain signaling cascades that drive unregulated cell division and proliferation^29^. Bioactive substances may also inhibit the malignant transformation by targeting pro-tumorigenic cells or the pro-metabolic carcinogen's conversion^30^.

Cancer cells acquire resistance to cancer treatments by several mechanisms, importantly mutated genes, proteins, enzymes and transcription factors. These mutated genes, enzymes, proteins and transcription factors are thought to be significant drug targets for slowing down the progression of cancer^31^. The signaling networks, such as genes, proteins, and enzymes, are shown in this perspective using graph theoretical network analysis. The graph theoretical concepts applied in the multifaceted signaling network, it may be possible to systematically examine the topology and functions of these selected network. Further, the graph theoretical network analysis can be used to predict the structural and dynamic properties of selected signaling pathway. Such predictions can assist the selection of ideal drug targets for reducing the complications of cancer^27^. It also gives data on the active site and molecular interactions of bioactive active compounds (ligands), which may help in the molecular docking study. As a result, the current research used pharmacoinformatics to examine the relevance of bioactive chemicals found in *Rasam* spices against oxidative stress-induced human malignancies. The predicted ADMET (absorption, distribution, metabolism, excretion, and toxicity) characteristics of the selected bioactive compounds were also investigated. Further, molecular dynamics simulation was investigated to determine the stability and binding modes of selected bioactive compounds with an appropriate cancer receptor protein.

## Materials and methods

### Graph theoretical network analysis

The graph theoretical network analysis was built using Cytoscape software version 3.7.1 and the Kyoto Encyclopedia of Genes and Genomes (KEGG) database^28^. The functions of numerous genes and proteins involved in the MAPK6/ERK3 (ko04657) in *Homo sapiens* was chosen to recognize the influential proteins.

### Protein preparation

The RCSB Protein Data Bank (PDB: http://www.rcsb.org/pdb) provided the X-ray crystallographic structure of MAPK6 (PDB ID: 7AQB)^32^. Prior to analysis, the protein was cleaned and missing residues were inserted using Swiss-PDB Viewer v4.1.0. The file was named target.pdb and saved for further analysis. We also utilized BIOVIA Discovery Studio Visualizer version 4.0 software (Accelrys Software Inc., San Diego, CA) in order to determine the protein structure and amino acid position from active regions, which was then utilized for molecular docking study.

### Active compounds retrieval and preparation

We found that around sixty-six bioactive components from twelve spices were used to make *Rasam*. The identified sixty-six components along with one known MAPK6 inhibitor drug were collected using the data repository (Indian Medicinal Plants, Phytochemistry, and Therapeutics (IMPPAT)) (https://cb.imsc.res.in/imppat/home), previously published studies^33^ and public database PubChem (https://pubchem.ncbi.nlm.nih.gov/).

### Binding site identification

A binding site in the target is a particular location on an enzyme/protein that permits the enzyme to attach to certain molecules and perform a chemical reaction. The major strategy to treat a disease is the binding of ligands or bioactive chemicals to the specific location of a protein/enzyme. This helps the bioactive chemicals to create enough contact sites in order to establish robust interaction with target enzymes by ensuring optimal and favourable catalytic areas. Using the Prank Web (https://prankweb.cz/) server, all possible active binding sites of targeted compounds were found for further analysis. Using the PyRx program, a receptor grid was created once the active site of the protein was selected.

### Molecular docking

Molecular docking approach is a crucial component of structural biology research, and it is one among the widely used technique in the process of drug design. The PyRx 0.8 tool^34^ and AutoDock Vina program^35^ was used to accomplish the molecular docking study. The ligand was one of the selected bioactive chemicals, and the receptor was MAPK6 (PDB ID: 7AQB). Polar hydrogen atoms and Kollman partial charges were introduced into the 3D structure using PyRx software. To compute docking energy affinities (kcal mol^−1^), the receptor and ligand files were stored in “.pdbqt” format. For each ligand, AutoDock Vina calculated the energy affinity values of up to ten different docking positions. AutoDock Vina effects were used to calculate each complex affinity energies based on the ligand conformation at the active binding site with RMSD between the original and subsequent structures taken into consideration. The amount of hydrogen bonds and non-covalent interactions for each complex were calculated using Discovery Studio Visualizer, which produced details, compounds, and interaction pictures (2D and 3D)^36^.

### Prediction of in silico pharmacokinetic and physicochemical properties

In silico prediction of ADME and physicochemical properties of the selected bioactive chemicals plays a major role in determining its integrity and efficiency. Selected bioactive chemicals into account, properties like molecular weight, molar refractivity, solubility, bioavailability, bioavailability radar plot, egg-boiled model, brain penetration, and human gastro intestinal absorption properties of the active bio-compounds have been determined using SwissADME (http://www.swissadme.ch/) webserver. The SwissADME webserver is a free tool that can predict the pharmacokinetic and drug-likeness properties of the test bioactive compounds^37, 38^.

### Toxicity prediction

Toxicity was predicted by determining the safety profile of the intended bioactive chemicals, which must have deadly effects on people and cause organ damage. As a result, the toxicity of the chosen bioactive chemicals was assessed using pkCSM-pharmacokinetics web-based server (http://biosig.unimelb.edu.au/pkcsm/prediction)^39^.

### Molecular dynamics simulation

The molecular dynamic simulation was evaluated to determine the binding stability, conformation and interaction modes between the selected bioactive compounds (ligands) and receptor (MAPK6). The selected ligand-MAPK6 complex files were subjected to molecular dynamics studies using GROMACS 2019.2 software^40–42^. The selected ligands topology was downloaded from PRODRG server^43^. The system preparation of all the complexes were as described earlier^24^. For molecular dynamic simulation, first vacuum was minimized using the steepest descent algorithm for 5000 steps. The complex structure was solvated in a cubic periodic box of 0.5 nm with a simple point charge (SPC) water model. The complex system was subsequently maintained with an appropriate salt concentration of 0.15 M by adding a suitable amount of Na^+^ and Cl^−^ counter ions. Each complex was allowed a simulation time of 50 ns from the NPT (Isothermal-Isobaric, constant number of particles, pressure, and temperature) equilibration was subjected in NPT ensemble for final run. The trajectory analysis of root means square deviation (RMSD) and root mean square fluctuation (RMSF) was performed in the GROMACS simulation package through the online server “WebGRO for Macromolecular Simulations (https://simlab.uams.edu/)”.

### Molecular mechanics Poisson–Boltzmann surface area (MMPBSA) calculation

The MMPBSA method was used to calculate the protein–ligand binding free energy of each complex. The free energy of binding was determined using the g_mmpbsa tool developed for GROMACS^24, 44^.

### Consent to participate

All authors agree to participate.

### Consent for publication

All authors agree for publication.

## Results

### Graph theoretical network analysis

**Figure 1:**
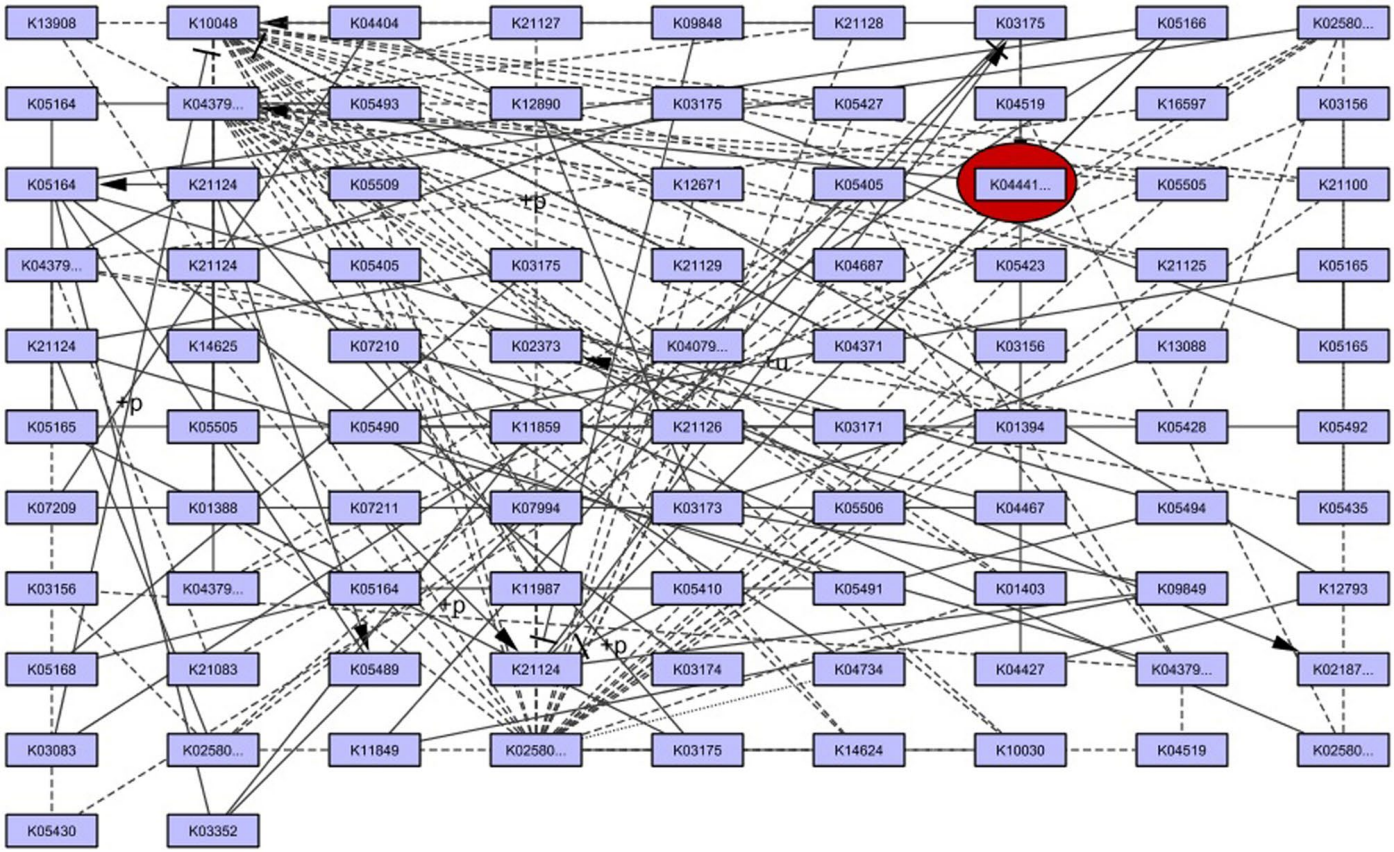
The signaling pathway of MAPK6/ERK3.

A graph was constructed for the MAPK6/ERK3 signaling pathway network. Numerous entities of genes, proteins (nodes) and their interactions (edges) in the current research work was displayed in Fig. 1. Based on centrality criteria such as degree, proximity, eccentricity, eigen vector, and radiality, the network has 92 nodes and 153 edges. The measured values of Betweennes (3483. 24) degree (2), closeness (0.004), eccentricity (0.125), eigen vector (0.0538), radiality (10.08), and stress (20,850) have shown the threshold value of all measures as well as significant node in the network (Tables 1, 2). The protein mitogen activated protein kinase 6 (MAPK6)/extracellular signal-regulated kinase 3 (ERK3) was identified as a potential drug target based on the centrality measure and its threshold values.

**Table 1:** The nodes and edges of influential protein (MAPK6).

Centrality measures	Maximum	Mean	Minimum
Betweenness	3483.24	467.19	13.34
Closeness	0.004	0.0017	0.0013
Degree	27	2.985	2
Eigenvector	0.0538	0.0247	0.0000000001
Eccentricity	0.125	0.0564	0.0625
Radiality	10.08	5.37	4.59
Stress	20,850	4529.82	114
Nodes	92		
Edges	153

**Table 2:** The results of threshold parameter values of the MAPK6 network analysis.

Gene	Betweenness	Closeness	Degree	Eccentricity	EIGENVECTOR	Radiality	Stress
K04441, K04440, K04371, K06855, K04464, K19603	52.33876592	0.003717	2	0.090909	0.053801959	9.747253	144
K07209,	103.6985941	0.002174	2	0.071429	8.95E-05	7.648352	156
K04427	471.5981179	0.003413	2	0.083333	0.006866011	9.483516	720
K04467	63.85047619	0.002137	2	0.0625	6.64E-04	7.56044	114
K03175	2009.804357	0.003922	6	0.090909	0.058181247	10.08791	14,254
K05405	13.34193734	0.003106	3	0.076923	0.142419691	9.351648	1088
K12793	334.5981179	0.002833	2	0.076923	8.11E-04	8.824176	486
K04404	196.8481179	0.002398	2	0.071429	1.05E-04	8.120879	298
K04734	253.4018821	0.002604	2	0.071429	0.04760016	8.483516	8984
K07210	116.4018821	0.002179	2	0.0625	0.005617381	7.659341	494
K02580, K04735,	560.0648877	0.002959	25	0.071429	0.403361797	9.175824	18,072
K04379, K09029, K04448, K04449, K04502	218.7644444	0.003333	25	0.076923	0.40408361	9.593407	792
K05494	1928.456998	0.003175	2	0.125	2.93E-04	9.241758	18,144
K05491	1918.882826	0.002747	3	0.111111	3.51E-05	8.703297	17,860
K03175	773.1666667	0.002597	2	0.090909	4.05E-05	8.659341	4284
K21124	1667.40129	0.003077	5	0.1	3.43E-04	9.318681	13,242
K21124	896.4314357	0.003534	6	0.1	0.008856044	9.78022	2316
K05164	3483.249418	0.003623	6	0.125	0.002486075	9.857143	32,586
K05166	1803.658925	0.004065	4	0.111111	0.012947182	10	20,850
K09849	457.7	0.003012	3	0.090909	0.001870239	9.054945	1648
K03173	308	0.002551	3	0.076923	2.24E-04	8.395604	1098
K10048	2756.21084	0.003745	27	0.083333	0.416220404	9.769231	34,478
K03083	610.4522186	0.003497	2	0.1	0.049949843	9.56044	10,184
K04371	610.4522186	0.003472	2	0.090909	0.049949843	9.538462	10,184
K03352	1252.614593	0.003636	3	0.1	0.007157894	9.681319	11,160
K11849	145.1490196	0.003215	2	0.083333	0.006989256	9.285714	856

### Retrieval of bioactive compounds and preparation

The accessible bioactive components of the selected spices (tamarind, red pepper, black pepper, cumin seed, fenugreek, asafoetida, garlic, tomato, coriander, curry leaves, sesame oil, and mustard) were searched using IMPPAT database. From the database, a list of sixty-six bioactive compounds were obtained from the twelve spices along with one standard MAPK6 inhibitor listed in Table 3.

**Table 3:** Bioactive compounds in *Rasam* ingredients and their binding affinity against MAPK6.

S. No	Ingredients	Compound id (CID)	Bioactive compound	Docking score (kcal mol^−1^)
1	Turmeric	969516	Curcumin	− 7.8
2		5469424	Demethoxycurcumin	− 7
3		5315472	Bisdemethoxycurcumin	− 7.7
4		10250249	5’-methoxycurcumin	− 6.7
5		443160	( +)-Alpha-Phellandrene	− 6.6
6		10429233	Dihydrocurcumin	− 7.2
7		92776	Zingiberene	− 6.2
8		10887971	( +)-Sabinene	− 6.6
9		64685	Borneol	− 5.2
10	Red pepper	1548943	Capsaicin	− 6.1
11		5281229	Capsorubin	− 8
12		5281228	Capsanthin	− 8.4
13		77994099	Bicyclomahanimbicine	− 9.5
14		107982	Dihydrocapsaicin	− 6.2
15		448438	Violaxanthin	− 9.4
16	Asafoetida	131751454	Assafoetidin	− 8.3
17		7067262	Franesiferol A	− 7.3
18		15559239	Franesiferol C	− 8.9
19		11892267	Conferol	− 9.2
20		12041593	Assafoetidinol A	− 9.8
21		636584	Assafoetidinol B	− 9.5
22	Cumin	95779	Thymohydroquinone	− 6
23		637563	*t*-anethole	− 6.1
24		6989	Thymol	− 6.8
25		5282799	Dihomolinoleic acid	− 5.2
26		985	Palmitic acid	− 5.1
27	Mustard	370	Gallic acid	− 5.8
28		5280343	Quercetin	− 8.1
29		5280805	Rutin	− 9.8
30		689043	Caffeic acid	− 6.4
31		445858	Ferulic acid	− 6.5
32		637542	*p*-Coumaric acid	− 6.6
33		1183	Vanillin gallic acid	− 6.1
34		65064	( −)-Epigallocatechin Gallate	− 8.9
35		107905	( −)-Epicatechin Gallate	− 9.5
36		442428	Naringin C	− 9.6
37		5281855	Ellagic Acid	− 8.3
38	Sesame oil	101746	Sesamolin	− 9.4
39		94672	Sesaminol	− 9.5
40		5281235	Beta-Cryptoxanthin	− 8.7
41		135404715	Hydroxysesamone	− 7.2
42		360837	2,3-epoxysesamone	− 7.8
43		72307	Sesamin	− 9.4
44	Black pepper	971	Oxalic acid	− 3.8
45		10364	Carvacrol	− 6.5
46		5318825	Koenigine	− 8.2
47		278055	Koenigicine	− 7.7
48		59053143	Murrayastine	− 7.5
49		398941	Dithymoquinone	− 3.3
50	Coriander	10282	Monoterpene	− 6.3
51		6549	Linalool	− 4.8
52		1549026	Geranyl acetate	− 5.3
53		6654	α-pinene	− 5.5
54	Tamarind	875	Tartaric acid	− 5.1
55	Garlic	65036	Allicin	− 4.4
56		16590	Diallyl disulfide	− 3.5
57		16315	Diallyl trisulfide	− 3.6
58		5386591	Ajoene	− 4.2
59		9793905	S-allyl-cysteine	− 4.4
60	Fenugreek	5570	Trigonelline	− 5.5
61		354616	Gentianine	− 6.1
62		444170	Fenugreekine	− 8.5
63		5280441	Vitexin	− 8.9
64	Tomato	446925	Lycopene	− 7.4
65		28523	Tomatine	− 10.6
66		54670067	Ascorbic acid	− 5.7
67	Standard MAPK6 inhibitor	447077	PD-173955 (6-(2,6-dichlorophenyl)-8-methyl-2-{[3-methylsulfanyl)phenyl] amino}-7H,8H-pyrido[2,3-d]pyrimidin-7-one)	− 9.3

### Binding site identification

**Figure 2:**
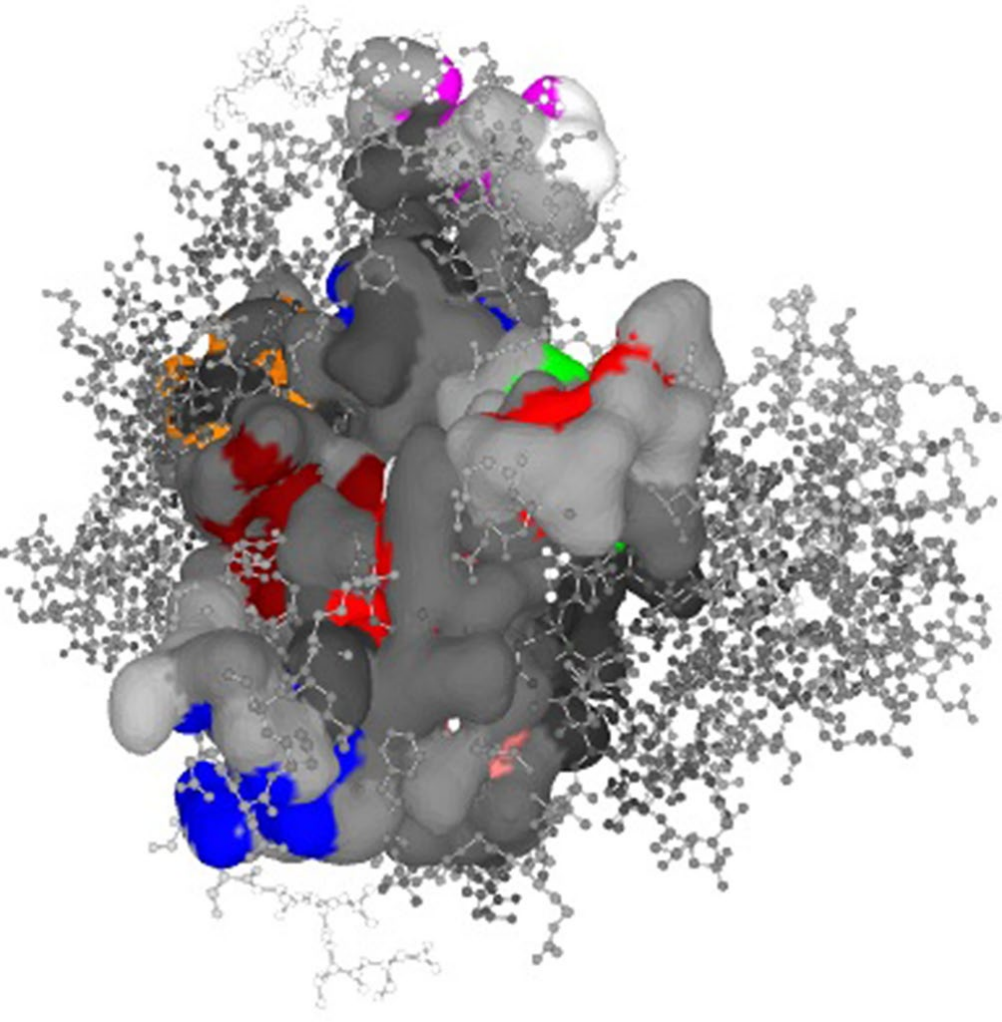
Showing active site and correspondence binding site of MAPK6 protein. Eleven binding pockets were predicted with different colour. Binding pocket 1 (17 AA with predicted score 9.16), pocket 2 (14 AA with predicted score 8.01), pocket 3 (15 AA with predicted score 4.75), pocket 4 (12 AA with predicted score 3.43), pocket 5 (13 AA with predicted score 3.10), pocket 6 (11 AA with predicted score 3.0), pocket 7 (10 AA with predicted score 2.14), pocket 8 (12 AA with predicted score 2.04), pocket 9 (08 AA with predicted score 1.90), pocket 10 (11 AA with predicted score 1.77) and pocket 11 (08 AA with predicted score 1.75).

Our evaluation of the crystal structure of MAPK6 (PDB: 7AQB) revealed the existence of 11 binding pockets, according to binding site analyses. The protein's recovered binding site residue are shown in the Fig. 2. Molecular docking investigations were also conducted using the obtained complex structure of the binding sites. Grid generation in molecular docking resulted in more reliable ligand posture scoring. As a result, we created a receptor grid for the selected MAPK6 protein based on the previously acquired binding site residues to achieve more precise scoring of our ligand poses. A receptor grid with a box dimensions of X = 38.6666, Y = 62.5914, and Z = 31.9740 in angstrom (Å) was created and utilized further for molecular docking experiments.

### Molecular docking

**Figure 3:**
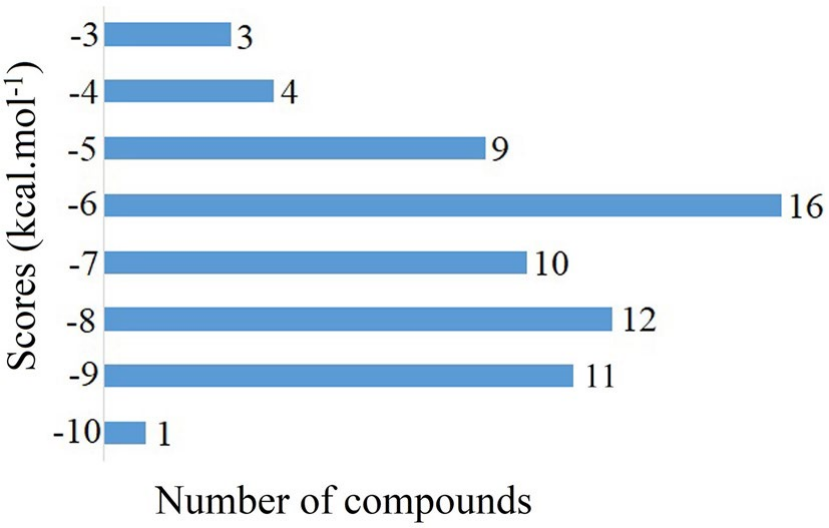
Showing the range of docking score distribution of sixty-six phytochemicals presence in the *Rasam*.

The optimum intermolecular interaction between the target protein and bioactive chemicals were investigated using molecular docking analysis. To analyse their binding capability, a specific number of bioactive chemicals (sixty-six) and known MAPK6 inhibitor were docked against MAPK6 using AutoDock Vina. Twelve bioactive chemicals were shown to have a lower binding energy (< − 9 kcal mol^−1^) with the target protein. The binding energy of the bioactive compounds following molecular docking was found to be scattered, ranging from − 3.50 to − 10.60 kcal mol^−1^, as illustrated in Fig. 3 and Table 3. The top four compounds (Assafoetidinol A (− 9.80 kcal mol^−1^), Naringin (− 9.60 kcal mol^−1^), Rutin (− 9.80 kcal mol^−1^), Tomatine (− 10.60 kcal mol^−1^)) were chosen for future research based on their binding energy with the amino acid residues in the active site of MAPK6. In this study, we used a standard MAPK6 inhibitor (CID: 447077) (− 9.30 kcal mol^−1^) as control, due to previously reported inhibitory activity against Bcr-Abl-dependent cell growth with an IC_50_ of 2–35 nM in different cell lines^45^.

### Interpretation of protein–ligand interactions

**Figure 4:**
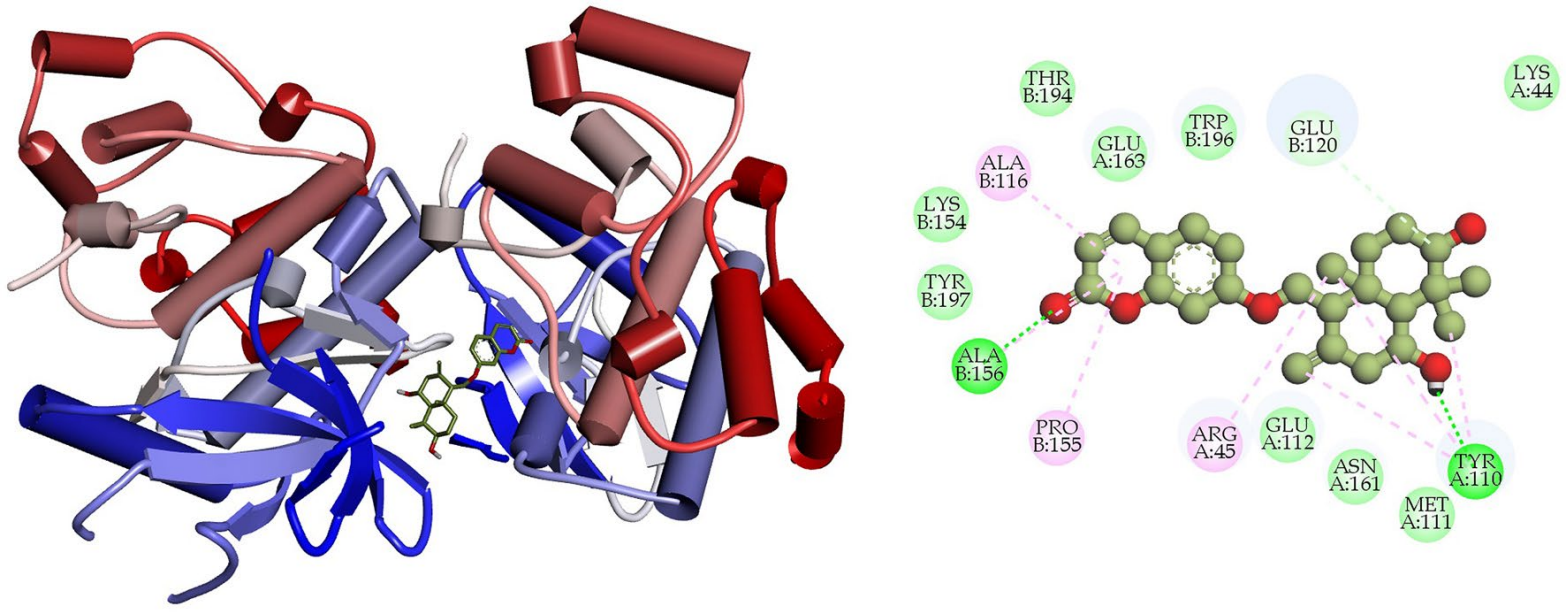
Depicted the interaction between the compound CID: 12041593 (Assafoetidinol A) and MAPK6. Left side representing 3D and the right side representing 2D complex protein–ligand interaction.

**Figure 5:**
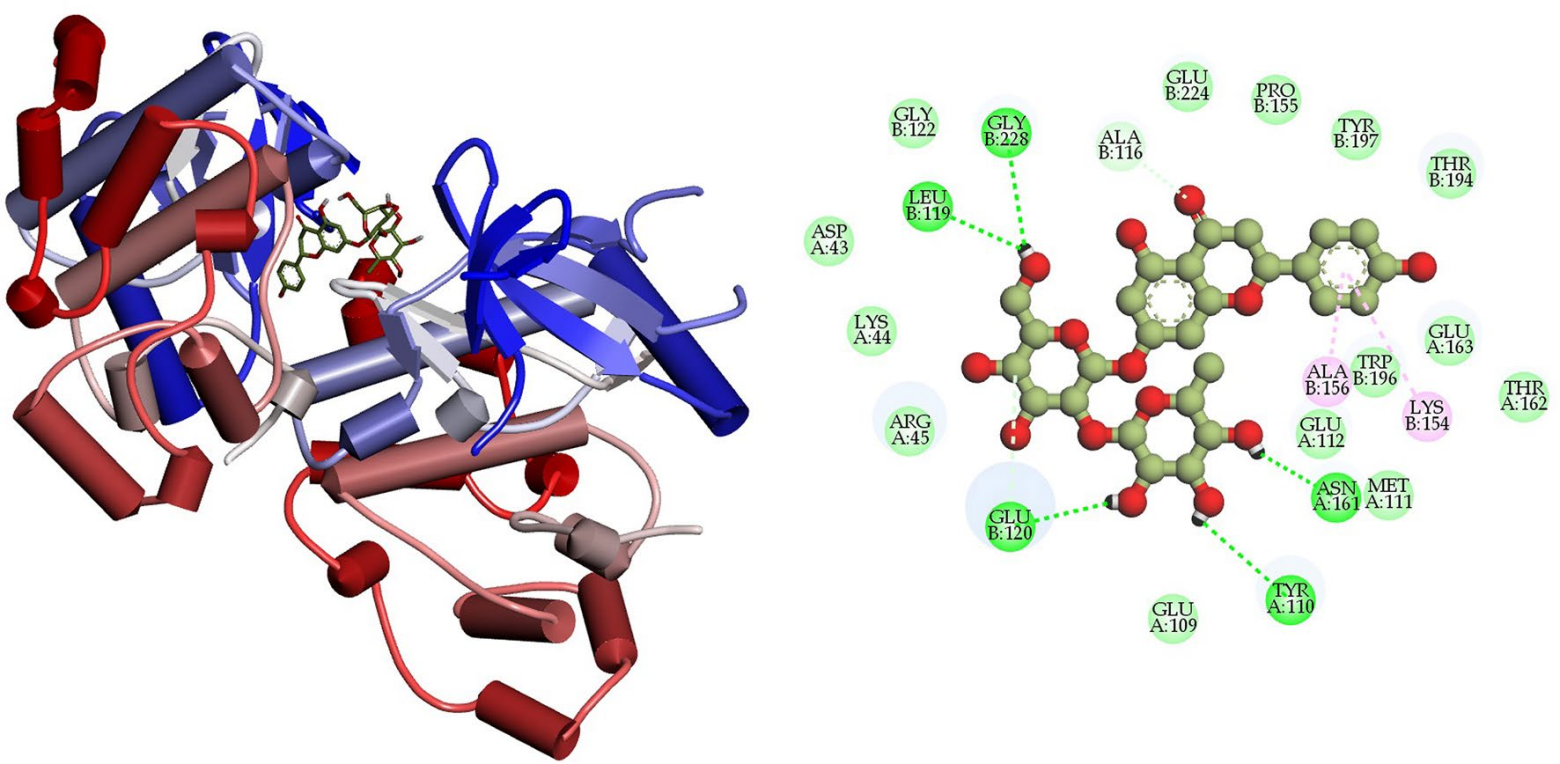
Depicted the interaction between the compound CID: 442428 (Naringin) and MAPK6. Left side representing 3D and the right side representing 2D complex protein–ligand interaction.

**Figure 6:**
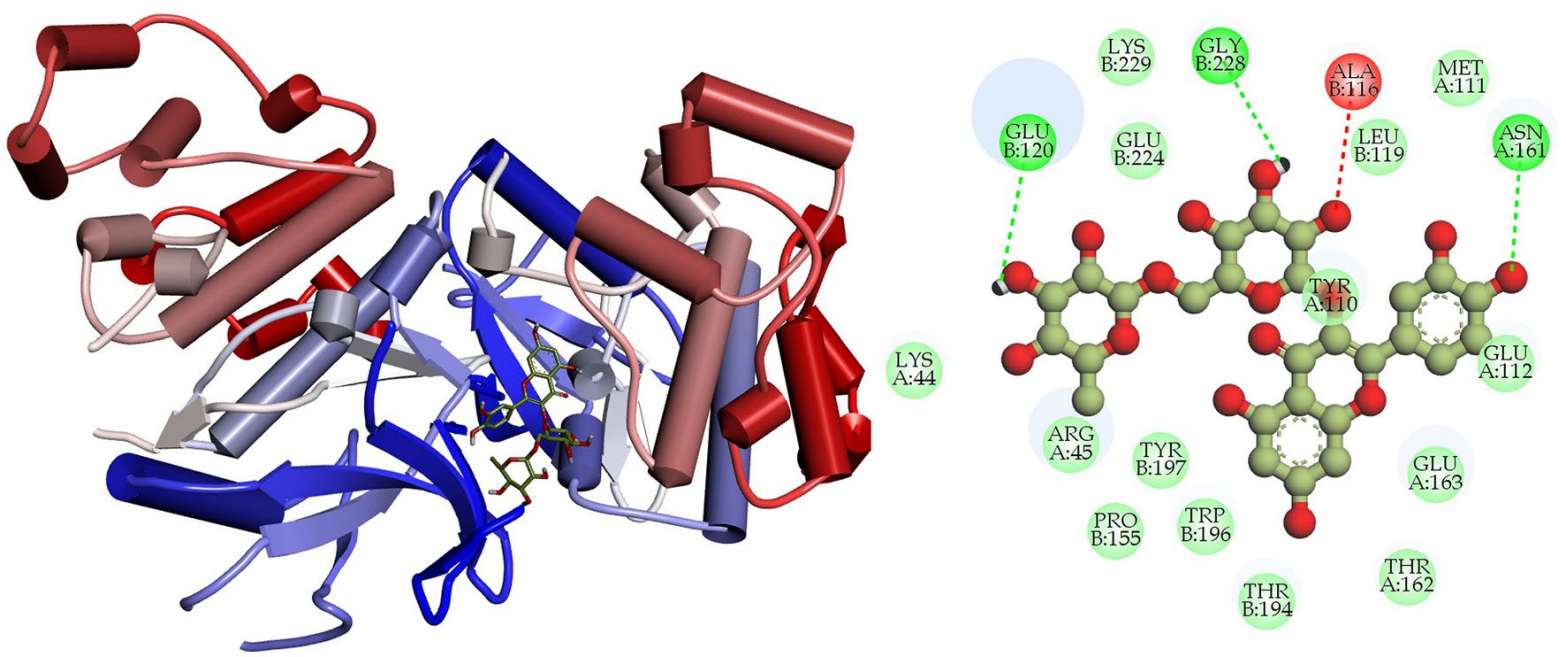
Depicted the interaction between the compound CID: 5280805 (Rutin) and MAPK6. Left side representing 3D and the right side representing 2D complex protein–ligand interaction.

**Figure 7:**
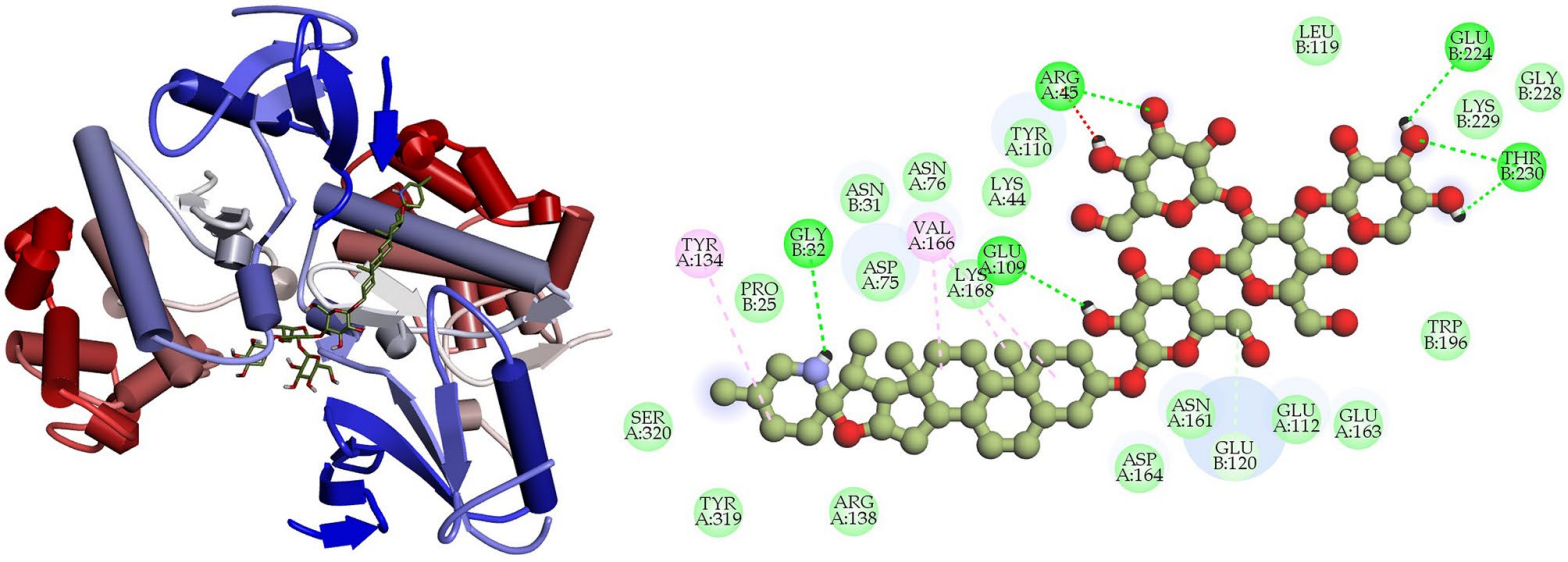
Depicted the interaction between the compound CID: 28523 (Tomatine) and MAPK6. Left side representing 3D and the right side representing 2D complex protein–ligand interaction.

**Figure 8:**
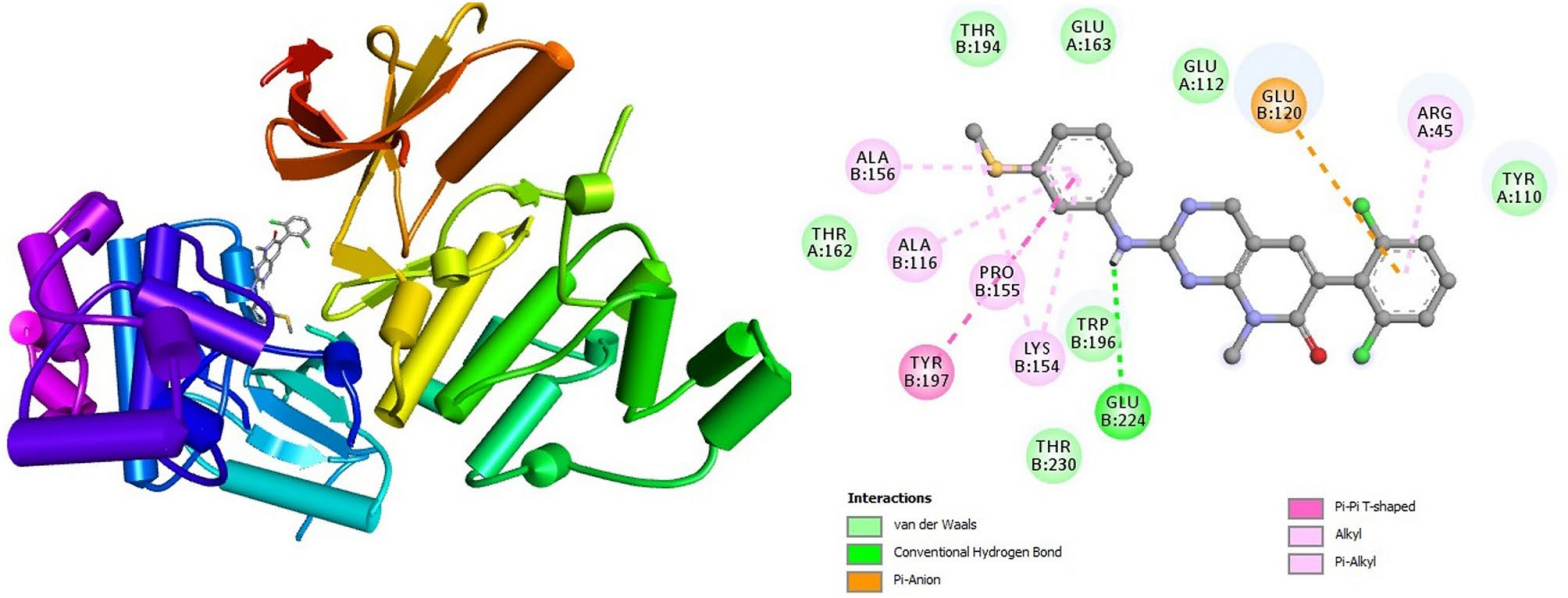
Depicted the interaction between the standard MAPK6 inhibitor (CID: 447077) drug and MAPK6. Left side representing 3D and the right side representing 2D complex protein–ligand interaction.

The interactions formed between the selected four ligands and MAPK6 has been visualized using BIOVIA Discovery studio visualizer tool. It was observed that compound CID: 12041593 (ASA) showed better interaction with MAPK6 and the binding energy was − 9.80 kcal mol^−1^. Compound CID: 12041593 formed four Van der Waals interactions with TYR110A (3.81 Å), GLU120 (3.57 Å), GLU163A (3.74 Å) and TRP196B (3.86 Å), two conventional and two carbon hydrogen bonds TYR110A (2.09 Å), ALA156B (2.10 Å) and GLU112A (3.58 Å), ASN161A (2.85 Å) respectively. Alkyl and Pi-Alkyl bond was also found at the position ARG45A (3.64 Å), ALA116B (3.75 Å), and PRO115B (3.67 Å), respectively showed in Fig. 4 and Table 4. For the compound CID: 442428 (NAR) it has been observed four hydrophobic and four hydrogen bonds with desired active site amino acid residues of MAPK6. CID: 442428 hydrophobic interactions with ALA116B (3.84 Å), LYS154B (3.76 Å), GLU163A (3.86 Å) and THR194B (3.75 Å), and four hydrogen bonds GLU120B (3.22 Å), LYS154B (3.11 Å), ASN161A (3.99 Å) and GLY228B (3.04 Å), respectively showed in Fig. 5 and Table 4. In the case of CID: 5280805 (RUT) exhibited four hydrophobic bonds with ARG45A (3.64 Å), GLU163A (3.59 Å), THR194B (3.71 Å) TRP194B (3.71 Å) and six hydrogen bonding with ARG45A (3.84 Å), ALA116B (4.03 Å), GLU120B (3.10 Å), ASN161A (3.20 Å), TYR197B (2.93 Å), GLU224B (3.44 Å) between active site amino acid residues of desired protein MAPK6, depicted in Fig. 6 and Table 4. For, CID: 28523 (TOM), it has observed two hydrophobic bonding with TYR134A (3.97 Å), VAL166A (3.75 Å), and four hydrogen bonding with GLY32B (3.42 Å), ARG45A (3.25 Å), ASN161A (4.08 Å), THR230B (3.25 Å) between the target protein, and showed in Fig. 7 and Table 4. For the standard MAPK6 inhibitor drug (CID: 447077) was observed five hydrophobic interactions with TYR110A (3.49 Å), LYS154B (3.67 Å), GLU163 (3.98 Å), THR194B (3.78 Å) and TRB196B (3.73 Å), one hydrogen bonding with TYR197B (3.42 Å) and one salt bridge GLU224B (3.68 Å) between the target protein, and showed in Fig. 8 and Table 4.

**Table 4:** List of bonding interactions between selected four bioactive compounds and standard MAPK6 inhibitor with MAPK6.

Compounds	Residues	Amino Acid	Distance (Å)	Bond category
CID: 12041593 Assafoetidinol A	110A	TYR	3.81	Hydrophobic
	120B	GLU	3.57	Hydrophobic
	163A	GLU	3.74	Hydrophobic
	196B	TRP	3.86	Hydrophobic
	110A	TYR	2.09	Hydrogen
	112A	GLU	3.58	Hydrogen
	156B	ALA	2.10	Hydrogen
	161A	ASN	2.85	Hydrogen
	154A	LYS	5.34	Salt
	45A	ARG	3.64	Alkyl
	116B	ALA	3.75	Alkyl
	155B	PRO	3.67	Pi-alkyl
CID: 442428 Naringin	116B	ALA	3.84	Hydrophobic
	154B	LYS	3.76	Hydrophobic
	163A	GLU	3.86	Hydrophobic
	194A	THR	3.75	Hydrophobic
	120B	GLU	3.22	Hydrogen
	154B	LYS	3.11	Hydrogen
	161A	ASN	3.99	Hydrogen
	228B	GLY	3.04	Hydrogen
CID: 5280805 Rutin	45A	ARG	3.64	Hydrophobic
	163A	GLU	3.59	Hydrophobic
	194B	THR	3.71	Hydrophobic
	196B	TRP	3.71	Hydrophobic
	45A	ARG	3.84	Hydrogen
	116B	ALA	4.03	Hydrogen
	120B	GLU	3.10	Hydrogen
	161A	ASN	3.20	Hydrogen
	197B	TYR	2.93	Hydrogen
	224B	GLU	3.44	Hydrogen
CID: 28523 Tomatine	134A	TYR	3.97	Hydrophobic
	166A	VAL	3.75	Hydrophobic
	32B	GLY	3.42	Hydrogen
	45A	ARG	3.25	Hydrogen
	161A	ASN	4.08	Hydrogen
	230B	THR	3.25	Hydrogen
CID: 447077 Standard MAPK6 inhibitor	110A	TYR	3.49	Hydrophobic
	154B	LYS	3.67	Hydrophobic
	163A	GLU	3.98	Hydrophobic
	194B	THR	3.78	Hydrophobic
	196B	TRP	3.73	Hydrophobic
	197B	TYR	3.42	Hydrogen
	224B	GLU	3.88	Salt bridge

### Pharmacokinetic and physicochemical properties prediction analysis

The ADME and physicochemical properties of selected bioactive compounds from *Rasam* were assessed through SwissADME (http://www.swissadme.ch/) webserver and these are presented in Table 5. From the assessed data in Table 5, the compound ASA (CID: 12041593; molecular weight 398.49 g mol^−1^) is alone found not to violate Lipinski’s rule of five. Other three compounds NAR (CID: 442428; molecular weight 580.53 g mol^−1^), RUT (CID: 5280805; molecular weight 610.52 g mol^−1^), TOM (CID: 28523; molecular weight 1431.59 g mol^−1^) and standard drug PD-173955 (molecular weight 443.35 g mol^−1^) violated Lipinski’s rule of five, due to higher molecular weight. The polar surface area of selected compounds was 79.90 Å^2^ (CID: 12041593), 225.06 Å^2^ (CID: 442428), 269.43 Å^2^ (CID: 5280805), 308.40 Å^2^ (CID: 28523) and 85.11 Å^2^. The observed results also demonstrated that CID: 12041593 and standard drug PD-173955 (CID: 447077) possesses a better human gastro intestinal (GI) absorption property, CID: 442428, CID: 5280805 and CID: 28523 have lower GI absorption properties. In general, higher GI absorption leads to enhanced bioavailability of the bioactive compound (CID: 12041593) of food (*Rasam*). Therefore, it might be better absorbed from the gastrointestinal tract upon oral administration. The higher number of H-bonds are possibly measured to be involved during protein ligand binding. From the result, the bioavailability score of four compounds showed better results (+ 0.55 for CID: 12041593 and standard MAPK6 inhibitor PD-173955, and + 0.17 for other three compounds) thereby relating with molecular properties CID: 12041593. Of the four compounds analyzed only ASA (CID: 12041593) and standard MAPK6 inhibitor (PD-173955) was predicted to have better chances as a possible drug-relevant candidate with anticancer potential. All the four bioactive compounds are soluble in nature, except standard drug (CID: 447077). The solubility class of all the compounds are listed in the Table 5. The synthetic accessibility score was found to be > 6 except CID: 12041593 and the standard drug (CID: 447077), which indicated that other three compounds are very difficult to synthesize.

**Table 5:** Pharmacokinetics and physicochemical parameters of selected bioactive compounds and standard MAPK6 inhibitor.

Parameter	CID: 12041593	CID: 442428	CID: 5280805	CID: 28523	CID: 447077
Formula	C_24_H_30_O_5_	C_27_H_32_O_14_	C_27_H_30_O_16_	C_50_H_85_NO_19_	C_21_H_16_C_l2_N_4_OS
MW (g mol^−1^)	398.49	580.53	610.52	1431.59	443.35
Num. heavy atoms	29	41	43	101	29
Num. arom. heavy atoms	10	12	16	12	22
Fraction Csp3	0.54	0.52	0.44	1	0.10
Num. rotatable bonds	3	6	6	20	4
Num. H-bond acceptors	5	14	16	29	21
Num. H-bond donors	2	8	10	15	1
Molar Refractivity	113.38	134.91	141.38	251.13	121.78
TPSA (Å^2^)	79.90	225.06	269.43	308.40	85.11
Solubility class	Moderately soluble	Soluble	Soluble	Moderately soluble	Poorly soluble
GI absorption	High	Low	Low	Low	High
BBB permeation	No	No	No	No	No
Violation of Lipinski’s rule of five	0	3	3	3	1
Violation of Veber rule	0	1	1	1	0
Bioavailability Score	0.55	0.17	0.17	0.17	0.55
Synthetic accessibility	5.16	6.16	6.52	10.00	3.07

**Figure 9:**
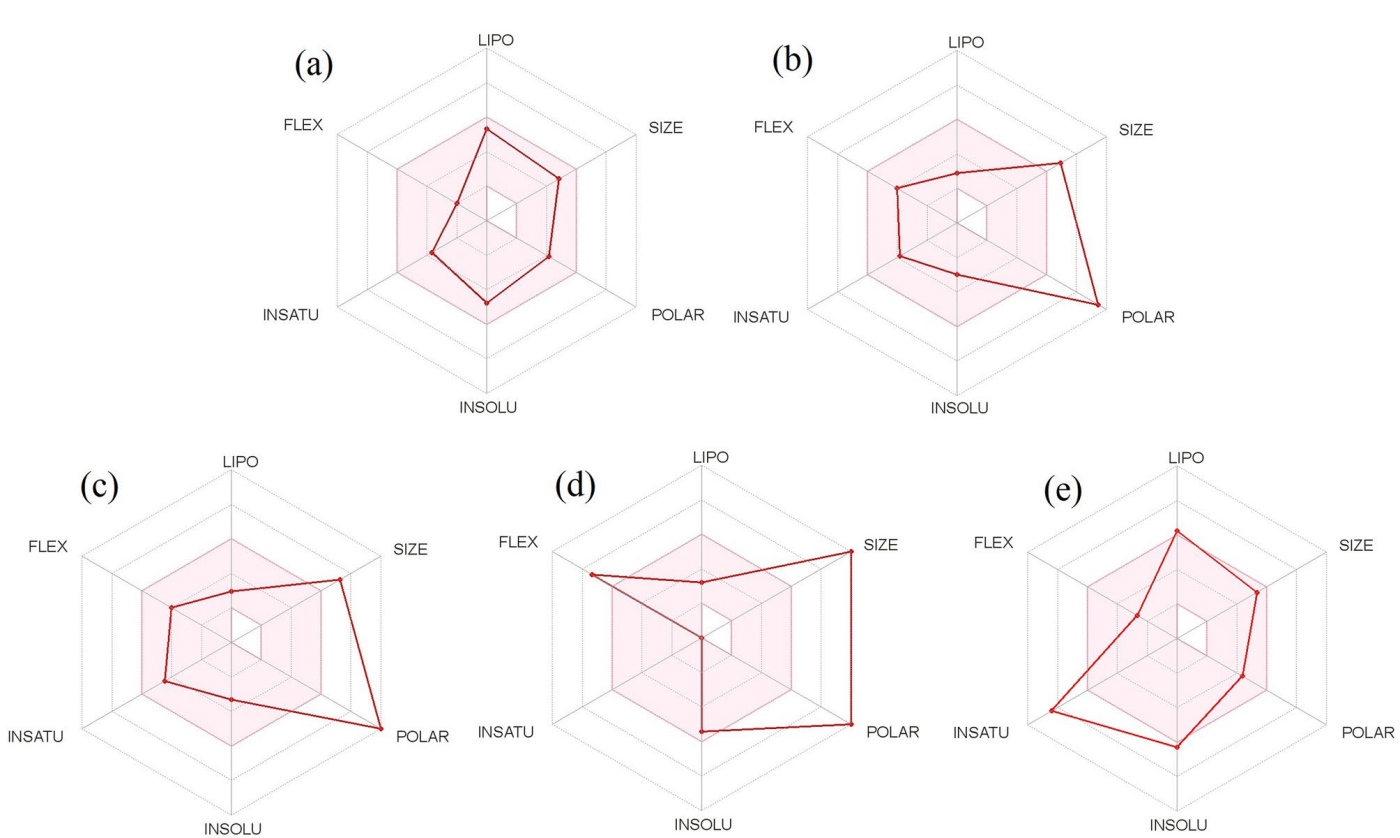
Bioavailability radar plot for oral bioavailability of selected bioactive compounds. CID: 12041593 (ASA) (**a**), CID: 442428 (NAR) (**b**), CID: 5280805 (RUT) (**c**), CID: 28523 (TOM) (**d**) and CID: 447077 standard MAPK6 inhibitor drug (**e**). The pink area exhibits the optimal range for each properties (Lipophilicity as XLOGP3 between − 0.7 and + 5.0; Size as molecular weight between 150 and 500 g mol^−1^; Polarity as TPSA (topological polar surface area) between 20 and 130 Å^2^; Insolubility in water by log S scale not higher than 6; Insaturation as per fraction of carbons in the sp3 hybridization not less than 0.25 and Flexibility as per rotatable bonds no more than 9).

The graphical representation of drug-likeness of four selected compounds are presented in the Fig. 9. The pink area within the hexagon represents the optimal range for the compounds. The recommended range for drug-like compound was insaturation (INSATU): fraction of carbons in the sp3 hybridization not less than 0.25, insolubility (INSOLU): log S not higher than 6, hydrophobicity (LIPO): between − 0.7 and + 5.0, rotatable bonds (FLEXI): no more than 9 rotatable bonds, molecular weight (SIZE): between 150 and 500 g mol^−1^ and polar surface area (POLAR): between 20 and 130 Å^2^). The compound CID: 12041593 (ASA) possess drug-like properties and are displayed by the red slanted hexagon within the pink shade (Fig. 9). Other three compounds (CID: 442428, CID: 5280805 and CID: 28523) and standard drug (CID: 447077) have high polar surface area, poor solubility and high molecular weight (size) which indicated off-shoot of the vertex (polar and size).

**Figure 10:**
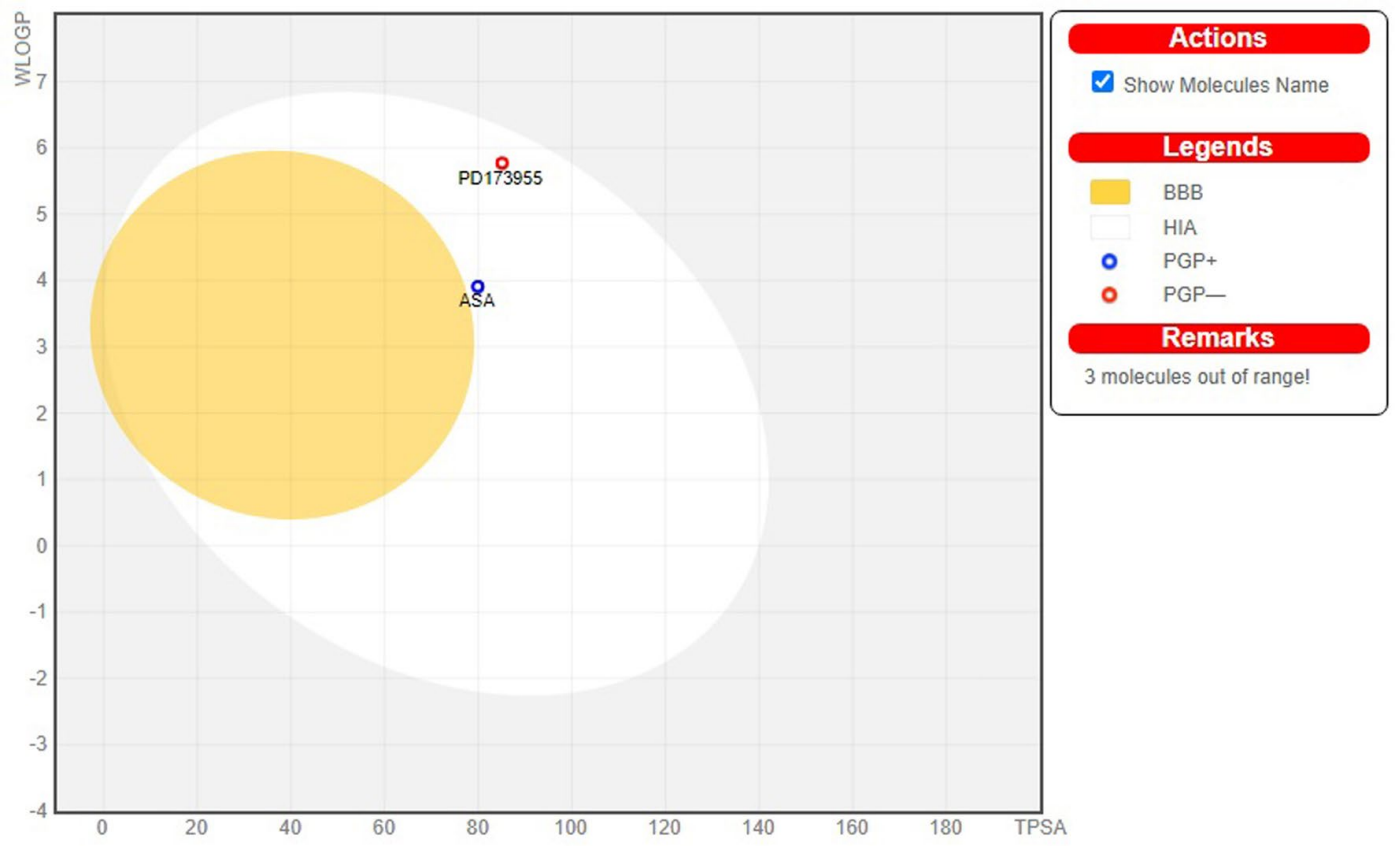
The EGG-BOILED model for the selected bioactive and standard MAPK6 inhibitor drug. The EGG-BOILED represents for intuitive evaluation of passive gastrointestinal absorption (HIA) white part and brain penetration (BBB) yellow part as well as substrates (PGP +) and non-substrates (PGP–) of the permeability glycoprotein (PGP) are represented by blue and red color circles, respectively, of the selected bioactive compound and standard MAPK6 inhibitor in the WLOGP-versus-TPSA graph. The grey region is the physicochemical space of compounds predicted to exhibit high intestinal absorption.

Moreover, the pharmacokinetic properties were investigated by using egg-boiled model of selected four compounds and standard MAPK6 inhibitor is depicted in Fig. 10. The egg-boiled model is helpful to predict two key pharmacokinetic properties simultaneously, i.e., the passive gastrointestinal absorption and blood brain barrier (BBB) penetration. The egg-shaped organisation plot shows that the compound present in the yolk (i.e. yellow region) represent the highly probable BBB permeation whereas albumin (i.e. white region) represent the highly probable human intestinal absorption. From the Fig. 10, the compound ASA (CID: 12041593) and standard drug (CID: 447077) found in albumin (white region) elucidated the good absorption in gastrointestinal region. Remaining three compounds were found to be outside of the boiled-egg region. From the above observed results, it can be interpreted that the compound ASA (CID: 12041593) have sufficient potential to be drug.

### Analysis of toxicity

In silico toxicity prediction of the selected four compounds has been performed using pkCSM-pharmacokinetics web-based server. The server has identified drug-induced hERG toxicity, AMES toxicity, LD_50_, hepatotoxicity, skin sensitization, *Tetrahymena pyriformis* (TP) toxicity, and minnow toxicity which was listed in Table 6.

**Table 6:** List of the drug-induced hERG inhibition, AMES toxicity, carcinogens, Tetrahymena pyriformis (TP) toxicity, rat acute toxicity (LD_50_ in mol/kg), and skin sensitisation along with Minnow toxicity of selected four compounds.

Compound ID	AMES toxicity	Max. tolerated dose (human)	hERG inhibition	LD50	Hepatotoxicity	Carcinogenicity	Skin Sensitisation	*T. pyriformis* toxicity	Minnow toxicity
CID: 12041593 Assafoetidinol A	No	− 0.448	No	2.889	Yes	No	No	0.651	1.212
CID: 442428 Naringin	Yes	0.491	No	2.652	No	No	No	0.285	4.999
CID: 5280805 Rutin	No	0.435	No	2.472	No	No	No	0.285	4.079
CID: 28523 Tomatine	No	0.216	No	2.482	No	No	No	0.285	13.574
CID: 447077 PD-173955	No	0.205	No	2.801	Yes	No	No	0.292	0.259

### Molecular dynamics simulation

Although, protein–ligand docking was widespread and has successful application, it just gives the static view of the binding pose of ligand in the active site of the receptor similar to a photographic image. Molecular dynamics (MD) must be employed to simulate the dynamics of atoms in the system as a function of time with integration of Newton’s equations of motions^46^. MD simulations for 50 ns were carried out for the top four receptor-ligand complexes obtained from the docking studies, that is 7AQB-ASA, 7AQB-NAR, 7AQB-RUT, 7AQB-TOM, 7AQB-STD and unbound apo form of the target MAPK6 protein (PDB ID: 7AQB) and their results were interpreted. To decipher the stability and fluctuations of these complexes, MD trajectories analysis was performed with the help of RMSD (Root Mean Square Deviation), RMSF (Root Mean Square Fluctuation), RG (Radius of gyration) and SASA (Solvent Accessible Surface Area) of receptor atoms.

**Figure 11:**
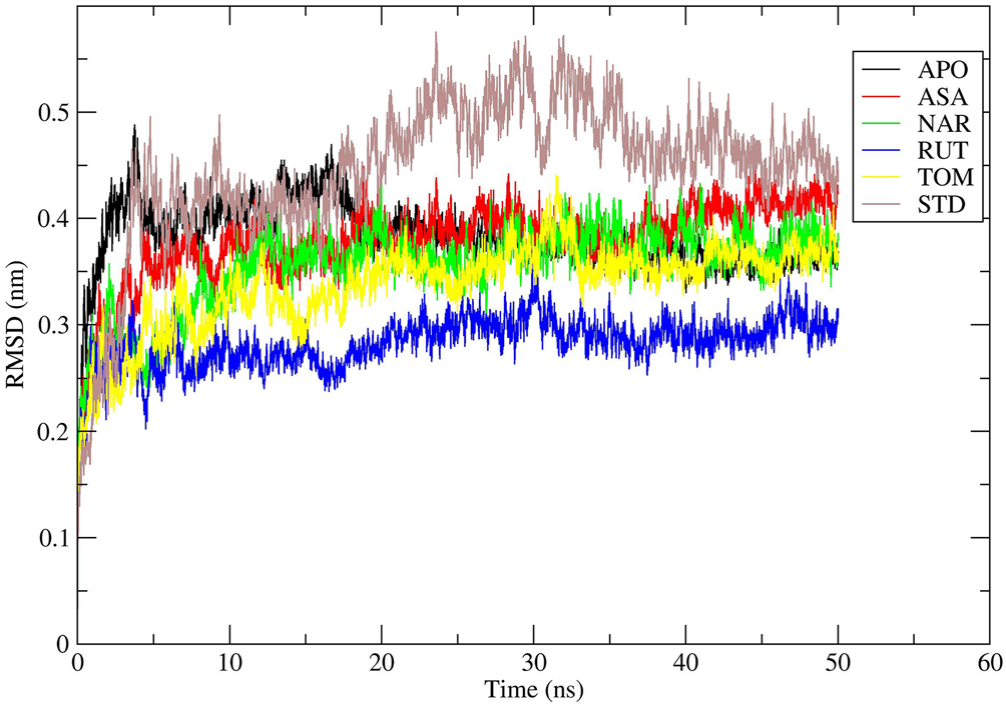
RMSD study plot for 50 ns MD Simulation of 7AQB-APO (Black), 7AQB-ASA (Red), 7AQB-NAR (Green), 7AQB-RUT (Blue), 7AQB-TOM (Yellow) and 7AQB-STD (Brown).

RMSD is an important parameter to analyse the equilibration of MD trajectories and check the stability of complex systems during the simulation process. RMSD of the protein backbone atoms were plotted against time to assess its variations in structural conformation. Initially, the 7AQB-ASA complex showed variations in backbone RMSD till 30 ns ranging from 0.15 to 0.44 nm. The stable conformation was attained in the time period between 21 and 50 ns with no considerable deviations in the values (Fig. 11). 7AQB-NAR complex showed variations in backbone RMSD till 20 ns ranging from 0.17 to 0.43 nm. The stable conformation was attained in the time period between 21 and 50 ns with no considerable deviations in the values (Fig. 11). The 7AQB-RUT complex showed variations in backbone RMSD till 30 ns ranging from 0.13 to 0.35 nm. The stable conformation was attained in the time period between 31–50 ns with no considerable deviations in the values (Fig. 8). The 7AQB-TOM complex showed variations in backbone RMSD till 35 ns ranging from 0.14 to 0.43 nm. The first stable conformation was attained in the time period between 36 and 50 ns with no considerable deviations in the values (Fig. 11). This clearly specifies that the protein underwent small structural changes in all the complexes during simulations.

**Figure 12:**
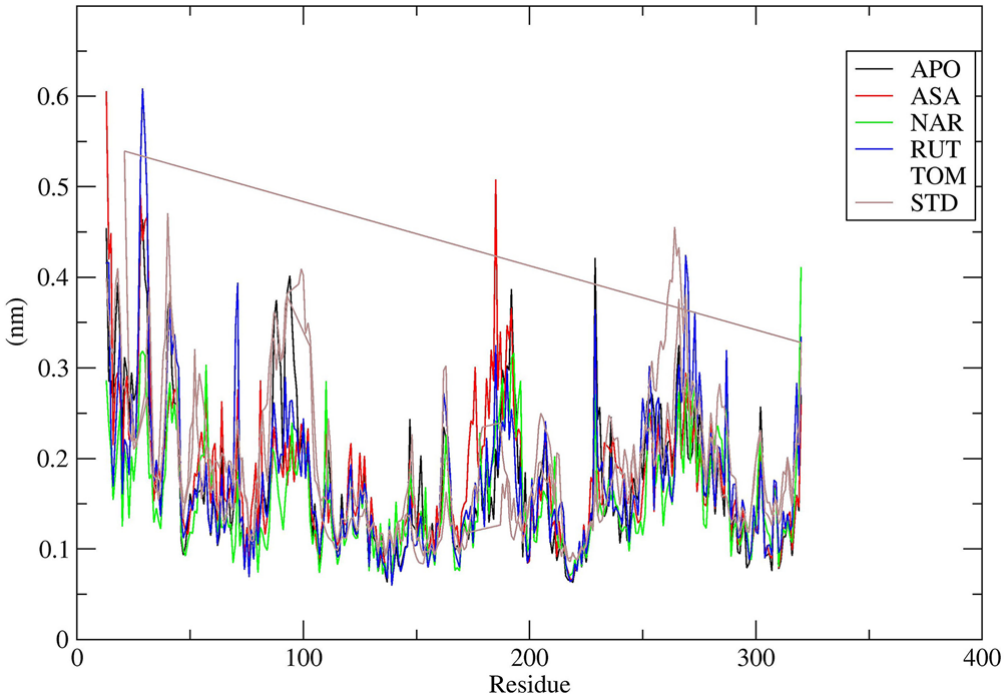
RMSF study plots for 50 ns MD Simulation. Chain of 7AQB-APO (Black), 7AQB-ASA (Red), 7AQB-NAR (Green), 7AQB-RUT (Blue7AQB-TOM (Yellow) and 7AQB-STD (Brown).

RMSF is an another crucial parameter while examining the stability and flexibility of complex systems during simulation^47^. RMSF was examined to analyse the changes in the behaviour of amino acid residues of target protein on binding to a ligand^48, 49^. The RMSF values for C**α** atoms of the protein were calculated and plotted with respect to the residues. In all the complexes, examined, the amino acid residues showed minimal fluctuations throughout the simulation. The amino acids of MAPK6 which interacted with ASA during docking showed minimal fluctuation values during MD simulation viz. CYS28, GLY29, LYS185 and LYS229, with NAR it showed low fluctuation values during MD simulation viz GLY29 and LEU192, with RUT showed minimal fluctuation values during MD simulation viz. GLY29, ARG70, LYS229 and ASN269 and with TOM it showed moderate fluctuation values during MD simulation viz*.* GLY29, LYS185, SER189, TYR266 and PRO301 (Fig. 12). These results revealed that binding of the ligands actuated no major effects on the flexibility of the protein.

**Figure 13:**
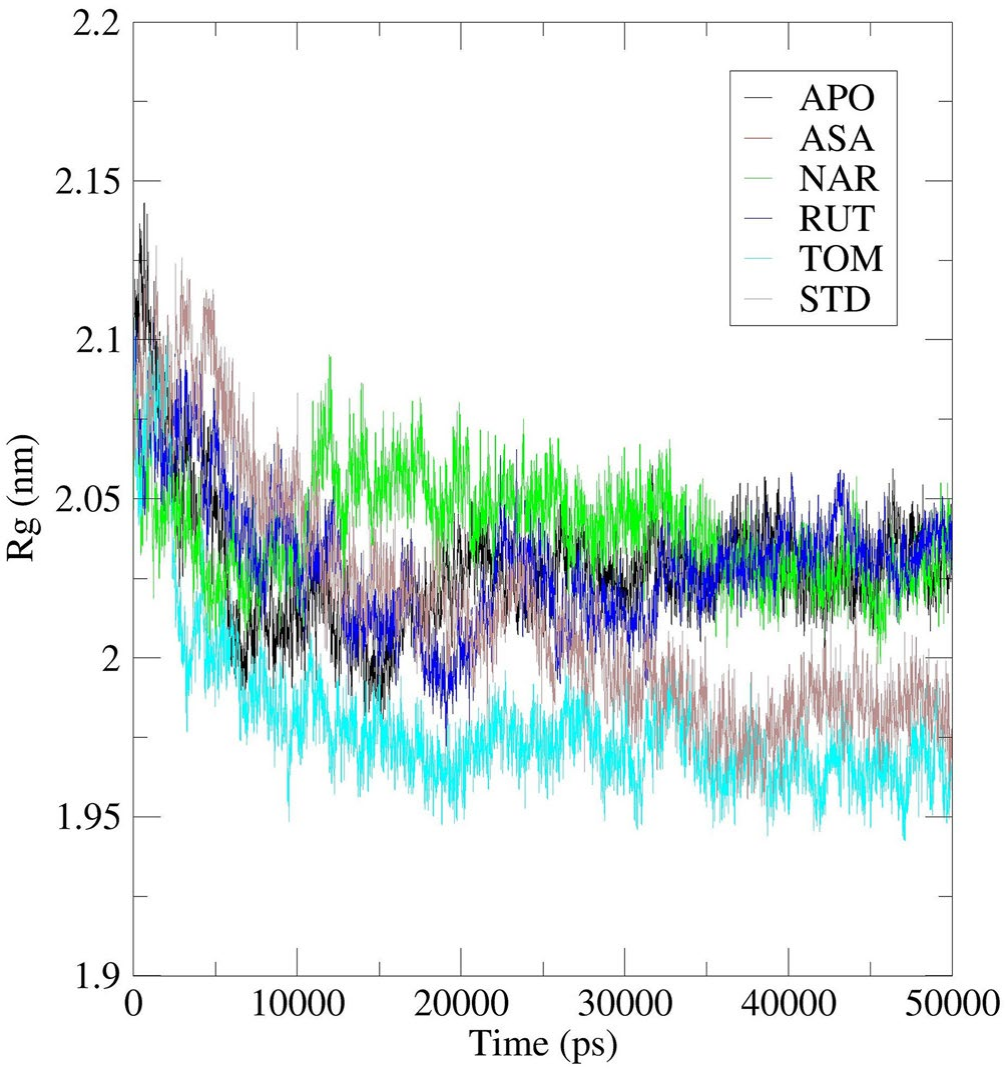
Radius of gyration study plot for 50 ns MD Simulation of 7AQB-APO (Black), 7AQB-ASA (Red), 7AQB-NAR (Green), 7AQB-RUT (Blue) 7AQB-TOM (Yellow) and 7AQB-STD (Brown).

Further, Radius of gyration (Rg) of the complex systems were also analysed. Rg is the root mean square distance of the atoms of the protein from the axis of rotation^49^. It is one among the important parameter that represents the overall change in the protein structure compactness and its dimensions during the simulation^50^. Higher Rg values characterize the protein as less compact and flexible while low values depict the high compactness and rigidity^47^. Rg values of backbone atoms of protein were plotted against time to examine the changes in structural compactness. Binding of ASA decreased the backbone Rg values till 30 ns. In the time period between 31 and 50 ns there were no considerable fluctuations and almost constant value of ~ 1.98 nm was maintained. Till end, the Rg values were found to be in the range between 1.95 and 1.99 nm. Complete analysis revealed that, in the initial stage the trajectory had shown its peak value of ~ 2.12 nm. Later this high value was never displayed again which shows the stability of protein in the complex (Fig. 13). Binding of NAR decreased the backbone Rg values till 15 ns. In the time period between 16 and 45 ns there were no considerable fluctuations and almost constant value of ~ 2.04 nm was maintained. Till end, the Rg values were found to be in the range of 2.02–2.05 nm. Complete analysis revealed that, in the initial stage, the trajectory showed its peak value of ~ 2.09 nm. Later, this high value was never displayed again which shows the stability of protein in the complex (Fig. 13). Binding of RUT decreased the backbone Rg values till 31 ns. In the time period between 32 and 50 ns there were no considerable fluctuations and almost constant value of ~ 2.03 nm was maintained. Till end, the Rg values were found to be in the range of 2.00–2.05 nm. Complete analysis revealed that, in the initial stage, the trajectory exhibited its peak value of ~ 2.10 nm. Later, this high value was never displayed again which shows the stability of protein in the complex (Fig. 13). Binding of TOM decreased the backbone Rg values till 10 ns. In the time period between 11 and 50 ns there were no considerable fluctuations and almost constant value of ~ 1.96 nm was maintained. Till end, the Rg values were found to be in the range of 1.94–2.00 nm. Complete analysis revealed that, in the initial stage, the trajectory had shown its peak value of ~ 2.10 nm. Later, this high value was never displayed again which shows the stability of protein in the complex (Fig. 13). The complete interpretation revealed that both the molecules induced no major structural changes in the protein.

**Figure 14:**
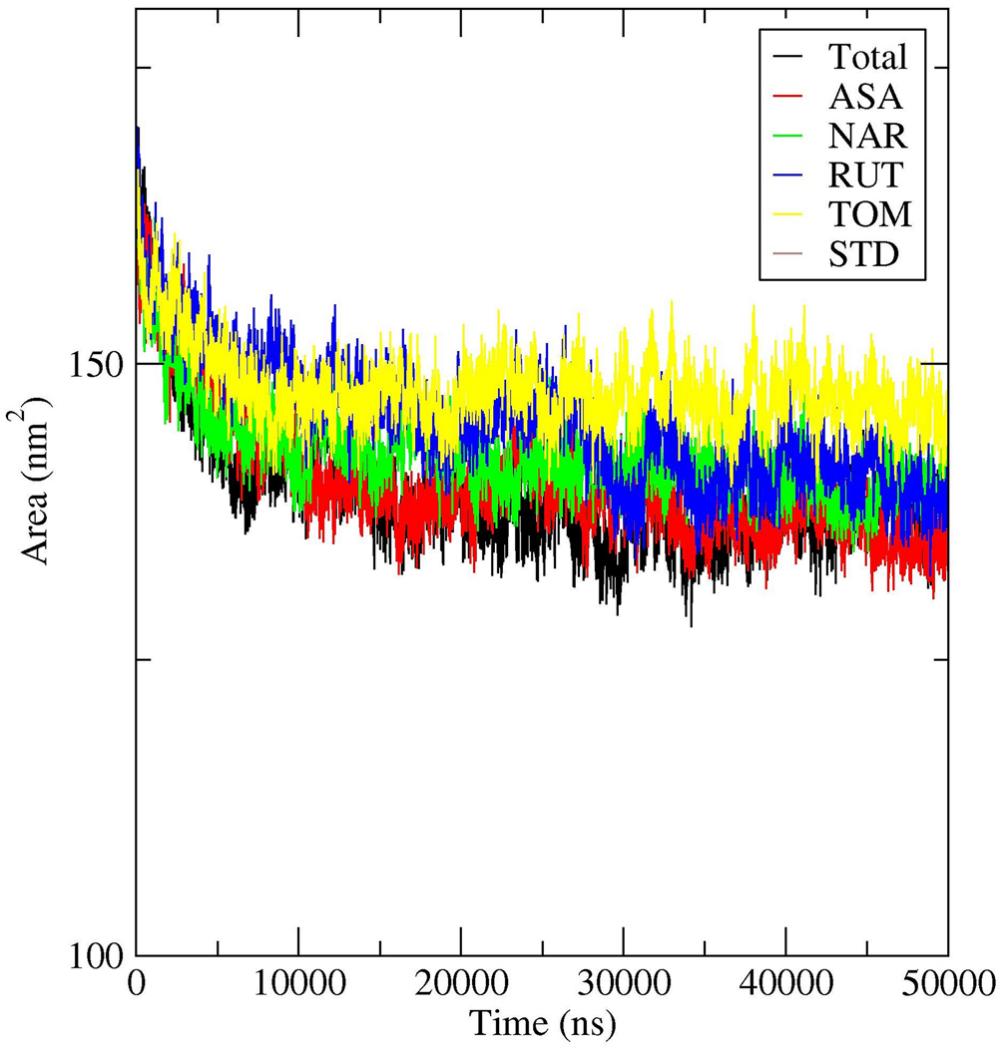
Solvent accessible surface area study plot for 50 ns MD Simulation of 7AQB-APO (Black), 7AQB-ASA (Red), 7AQB-NAR (Green), 7AQB-RUT (Blue) 7AQB-TOM (Yellow) and 7AQB-STD (Brown).

Moreover, analysis of Solvent Accessible Surface Area (SASA) for all the complexes was implemented. SASA is the substantial criterion to examine the extent of exposure of receptor to the surrounding solvent molecules during simulation^47, 51^. In general, binding of ligand may induce the structural changes in the receptor and hence the area in contact with the solvent also may vary^49^. SASA values of protein was plotted against time to estimate the changes in surface area. For SASA complex, the trajectory showed decrease in the values till 15 ns. Except few time intervals, minute fluctuations were observed throughout the simulation period (Fig. 14). The average SASA value was found to be ~ 138 nm^2^ and were in the range of 150–130 nm^2^. For NAR complex, the trajectory showed decrease in the values till 10 ns. Except few time intervals, minute fluctuations were observed throughout the simulation period (Fig. 14). The average SASA value was found to be ~ 142 nm^2^ and were in the range of 149–134 nm^2^. For RUT complex, the trajectory showed decrease in the values till 10 ns. In the time interval of 11–28 ns, minute fluctuations were observed and from 29 to 34 ns a moderate fluctuation was observed (Fig. 14). The average SASA value was found to be ~ 140 nm^2^ and were in the range of 154–133 nm^2^. For TOM complex, the trajectory showed decrease in the values till 10 ns. Except few time intervals, minute fluctuations were observed throughout the simulation period (Fig. 14). The average SASA value was found to be ~ 148 nm^2^ and were in the range of 154–140 nm^2^. Overall, the analyses revealed that the surface area of protein in complexes were shrunken during the simulation.

**Figure 15:**
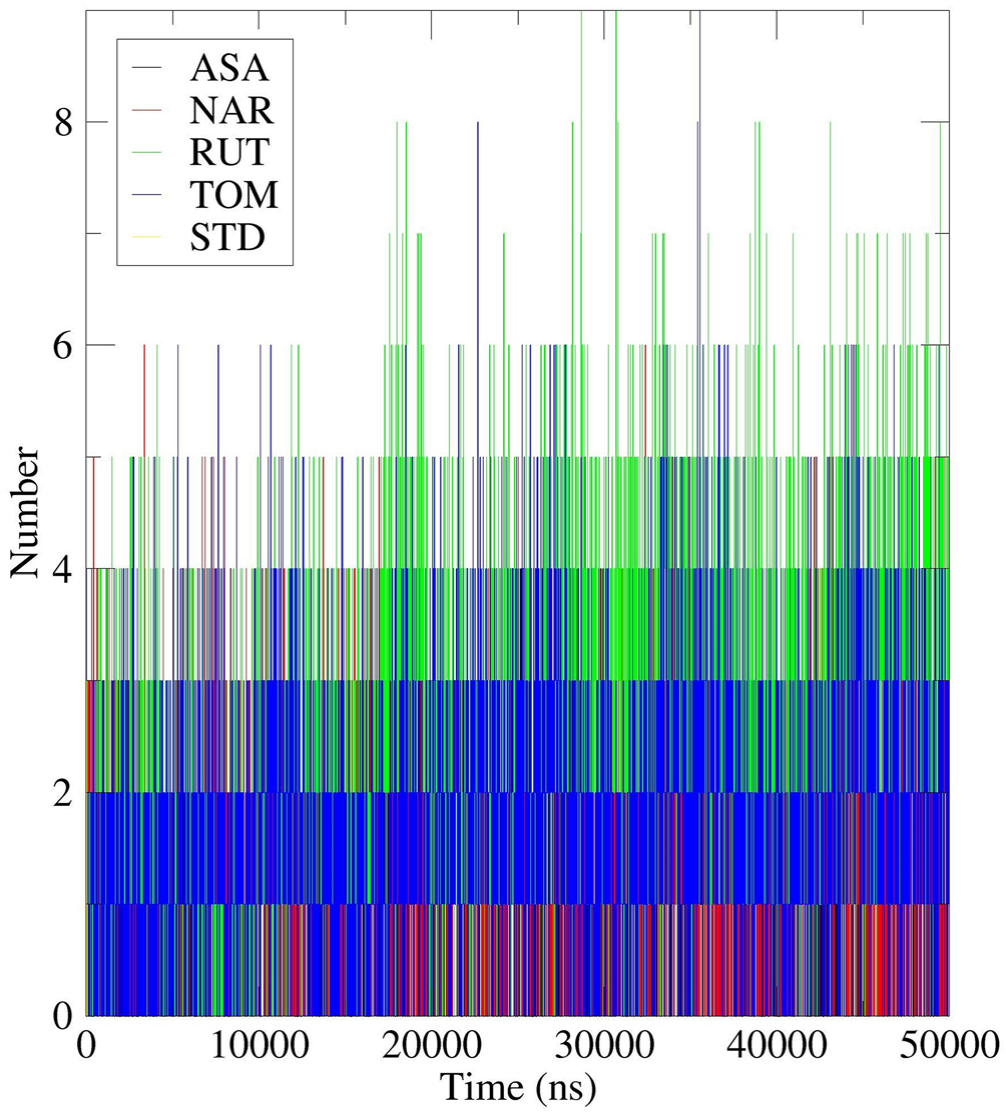
Intermolecular hydrogen bonding study plot for 50 ns MD Simulation of 7AQB-APO (Black), 7AQB-ASA (Red), 7AQB-NAR (Green), 7AQB-RUT (Blue) 7AQB-TOM (Yellow) and 7AQB-STD (Brown).

To examine the binding energy of the ligands with the target protein, the MD trajectories were analyzed to interpret the extent of hydrogen bond formation during the entire course of simulation and was depicted in Fig. 15. SASA had formed good number of H-bonds with the receptor protein with a maximum of five bonds at several time frames indicating the stronger affinity towards the target. Consistency was maintained in forming almost two hydrogen bonds for the entire simulation time which signifies the stability of the complex. For the NAR complex, the consistency was maintained in forming three hydrogen bonds with maximum of six bonds at certain time periods. For RUT complex, the consistency was maintained in forming four hydrogen bonds with maximum of nine bonds at certain time periods. For the TOM complex, the consistency was maintained in forming two hydrogen bonds with maximum of nine bonds at certain time periods. This clearly signifies that the top phytochemicals have the stronger affinity with the target protein.

We also analyzed the binding interaction of 7AQB-ASA, 7AQB-NAR, 7AQB-RUT, 7AQB-TOM and 7AQB-STD complexes at a time interval of 10 ns (Supplementary 1). For 7AQB-ASA complex ARG70, ILE83 and ASP93 amino acids, for 7AQB-NAR complex LYS67, ARG70 and PHE81 amino acids, for 7AQB-RUT complex HIS61, ARG151 and HIS187 amino acids, for 7AQB-TOM complex LEU33, VAL34 and LYS49 amino acids and for 7AQB-STD complex GLU112 and TRP196 amino acids were found to have constant interaction during the entire period of simulation (Supplementary file 2).

### Binding free energy analysis/molecular mechanics Poisson–Boltzmann surface area (MM/PBSA)

For the last 20 ns (30–50 ns) of simulation trajectories with dt 1000 frames, the binding free energy (ΔG bind) was calculated for 7AQB-ASA, 7AQB-NAR, 7AQB-RUT, 7AQB-TOM and 7AQB-STD complexes by utilizing MM/PBSA method. ΔG bind of 7AQB-ASA, 7AQB-NAR, 7AQB-RUT, 7AQB-TOM and 7AQB-STD complexes were − 192.310 ± 26.072 kJ mol^−1^, − 189.432 ± 25.360 kJ mol^−1^, 199.407 ± 32.354 kJ mol^−1^, − 213.069 ± 28.252 kJ mol^−1^ and 187.134 ± 33.329 kJ mol^−1^ respectively. These high negative values of free binding energies signify that four test ligands and standard have high binding affinity towards MAPK6.

## Discussion

The purpose of this research work was to look at the cancer-preventive impact of bioactive chemicals found in the south Indian cuisine *Rasam* by using graph theoretical network and pharmacoinformatics analysis. Pharmacoinformatics is a collection of in silico molecular modeling tools for screening the bioactive substances based on their binding affinities, pharmacokinetics, and pharmacodynamic features^52^. By enabling researchers to narrow down the biological and synthetic research impacts, pharmacoinformatics has sped up the discovery of bioactive substances. Several substances have their positive effects predicted using pharmacoinformatics research, which were then validated by in vitro and in vivo activities. Understanding how chemicals bind, interact, and inhibit/stimulate a certain protein might help researchers find therapeutic options for certain disease conditions.

Initially, a graph theoretical network was developed using centrality metrics, and it was suggested for metabolic networks that included enzymatic cascades and synergistic ligand-enzyme interactions. Biological networks, which are made up of a number of vertices (or nodes) linked in a pattern by a set of edges (or connections), are designed to mimic the structure of genuine biological systems. MAPK6/ERK3 was identified as a receptor (target) for ligand (bioactive substances) binding in ROS-induced oxidative stress that leads to malignancies, according to the network analysis study. MAPK6 pathway has been shown to be impacted not just by receptor ligand interactions, but also by various other stresses. MAPK6 is shown to be a key factor for organogenesis, cancer cell growth and invasiveness. MAPK6 overexpression has been detected in numerous human cancers, including squamous cell lung carcinoma, oral squamous cell carcinoma, gastric cancer, breast cancer and melanoma^53^. DNA microarray studies have produced inconsistent information about the regulation of MAPK6 expression in cancer. Several investigations demonstrated that expression of MAPK6 mRNA is down-regulated in brain tumors, ovarian carcinoma and cutaneous melanoma, and up-regulated in leukemias, adrenocortical carcinoma, squamous cell lung carcinoma, salivary adenoid cystic carcinoma, tongue squamous cell carcinoma and cervical cancer^54^. Furthermore, since MAPK signaling cascade regulate both mitogen- and stress-activated signals, the regulation of both pathways by ROS has drawn researchers’ attention^55^. The goal of the current research work was to look into the detoxification/neutralization of ROS by employing bioactive chemicals found in *Rasam* spices to protect cells against cancer. A total of sixty-six bioactive compounds were chosen from twelve spices using the IMPPAT database, as well as previously published publications on their effects against different human malignancies. All the chemicals chosen were docked against MAPK6 protein kinases, with binding energy ranging from − 3.50 kcal/mol^−1^ to − 10.60 kcal mol^−1^. Four compounds (Assafoetidinol A (− 9.80 kcal mol^−1^), Naringin (− 9.60 kcal mol^−1^), Rutin (− 9.80 kcal mol^−1^), Tomatine (− 10.60 kcal mol^−1^)) and PD-173955 (CID: 447077) (− 9.30 kcal mol^−1^) have been chosen for future analysis based on their significant binding ability, strong hydrophobic and hydrogen bonding interactions with amino acid residues present in the active site of MAPK6 protein. Similarly, Ahammad et al. identified seventy phytochemicals from Neem (*Azadiractha indica*) plant, and screened against MCM7 protein by using the molecular docking simulation tool. The binding affinities found after molecular docking of the phytochemicals compound have shown a distributed range between − 3.1 and − 9.0 and kcal mol^−1^^56^.

Bioactivity of a substance is largely governed by its absorption, distribution, metabolism, and excretion (ADME) characteristics, all of which are connected to its pharmacokinetic characteristics. The bioavailability of dietary phytochemicals to target cells, as well as their absorption and metabolism in the human body are certain key aspects in promoting their bioactivity and maintaining body health^57^. The small intestine absorbs some, but not all, of the components of dietary phytochemicals into the circulatory system. Some phytochemical compounds that were absorbed by the colon and altered by the gut microbiota; the microbial metabolites that were released back into the circulation showed significant activity^58^. In order for any molecule to permeate the membrane, phytochemicals/test substance must break hydrogen bonds in the aqueous environment and partition across the membrane^59^. The polar surface area (PSA) of a chemical is connected to its hydrogen-bonding potential, whereas molecular mass and lipophilicity are associated to membrane permeability^60^. As a consequence, the ADME properties must be assessed at the earlier stages of drug design and discovery process in order to pass the standard clinical studies required to be considered as prospective therapeutic candidate^61^. In this study, all the discovered phytoconstituents were confirmed in terms of usual pharmacokinetic properties using multiple bioinformatics methods. Phytochemicals are naturally derived from variety of plants that are often consumed by humans and are usually considered safe to consume. While most phytochemicals are not regulated by the Food and Drug Administration (FDA) in the United States, their potential toxicity is unknown.

Phytochemicals are utilized as supplements in conjunction with illness therapy all around the world, but the users do not necessarily inform this to their physicians^62^. Substance toxicity refers to the property of any compound to be poisonous and to cause harm to an organism. Toxicity testing of a substance necessitates in vitro and in vivo animal experiments, which is a time-consuming, expensive, and complicated technique. Because there are no animal trials, and improved precision, accessibility, and speed, in silico toxicity assessment has become very popular in recent times and it can offer information on any synthetic or natural molecule. In this work, in silico approaches were used to estimate the toxicity levels of four chemicals. The non-carcinogenic and non-skin irritating properties of four substances were determined using in silico testing. Three compounds, Assafoetidinol A, Rutin, and Tomatine were shown to be negative in Ames testing. Toxicity tests revealed that the four phytochemicals chosen had no negative side effects (hERG). The LD_50_ (median fatal dosage) indicates the immediate or acute toxicity of substances that were determined to be the most effective in the investigation. Hence, the complexes of these compounds were subjected to molecular dynamics simulations and the results were analysed with the results of apo form of MAPK6 (7AQB). The complexes were validated by interpreting the RMSD, RMSF, Rg, SASA and the lead phytochemical complexes were found to be stable during the simulations.

## Conclusion

Traditionally, home-cooked meals have been shown to help avoid chronic illnesses, improve health, and save treatment costs while also boosting quality of life. This study looked at the antioxidant properties of bioactive chemicals found in the south Indian cuisine, *Rasam* against oxidative stress-induced human malignancies. In the human body, ROS is a metabolic by-product of cellular respiration. Oxidative stress and overexpression of MAPK6 protein are caused by an increase in ROS levels. MAPK6 overexpression causes a cascade of events in cells, including mutations and carcinogenesis. Through a thorough pharmacoinformatics-based molecular docking investigation of bioactive substances against MAPK6, the antioxidant potential of *Rasam* has been proven in the current work. In silico molecular docking investigations found that the four lead phytochemicals (Assafoetidinol A, Naringin, Rutin, and Tomatine) may suppress MAPK6/ERK3 expression. In addition, MD stimulation tests and in silico pharmacokinetic prediction analyses gives the safety profile of four lead compounds as well as the stability of the protein–ligand complex, although, in order to determine the *Rasam*’s effectiveness, further in vitro and in vivo animal research work will be necessary.

## Supplementary Information

Supplementary Information 1.

Supplementary Information 2.
