# Supplementary material for: Pharmacoinformatics-based investigation of bioactive compounds of Rasam (South Indian recipe) against human cancer
Source: Sci Rep. 2021 Nov 2;11:21488. doi: 10.1038/s41598-021-01008-9 (PMC8563928; doi:10.1038/s41598-021-01008-9)
Supplement: Supplementary file 1 — Supplementary Information 1. [file 41598_2021_1008_MOESM1_ESM.doc]

**Supplementary**

7AQB-ASA, 7AQB-NAR, 7AQB-RUT, 7AQB-TOM and 7AQB-STD complexes interaction at various time interval of MD simulation (10 ns, 20 ns, 30 ns, 40 ns, 50 ns)

| 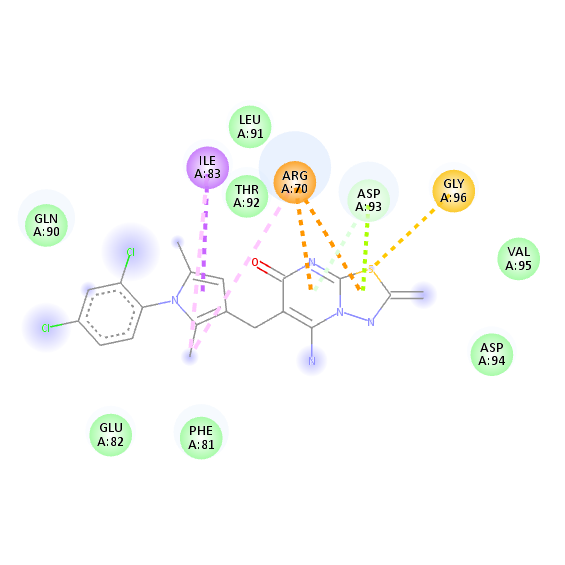 | 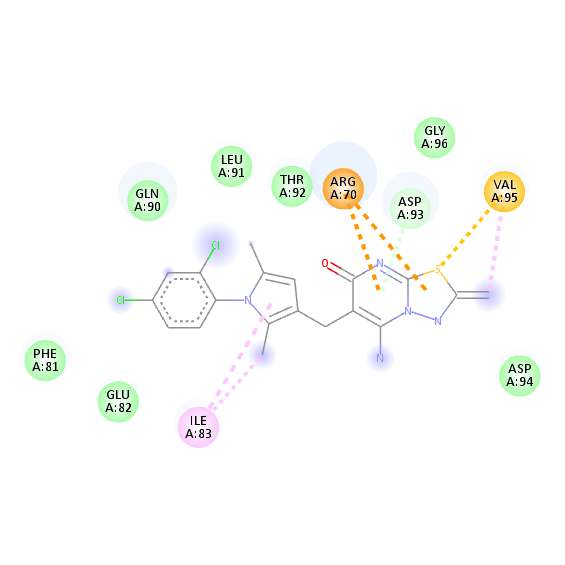 |
| --- | --- |
| ASA 10 ns | ASA 20 ns |
| 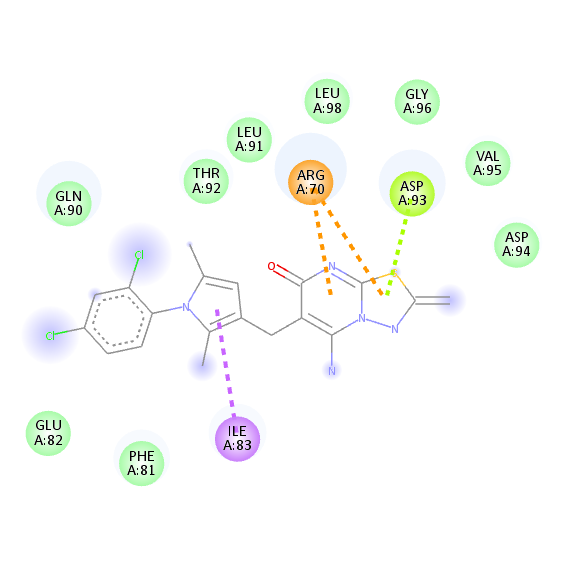 | 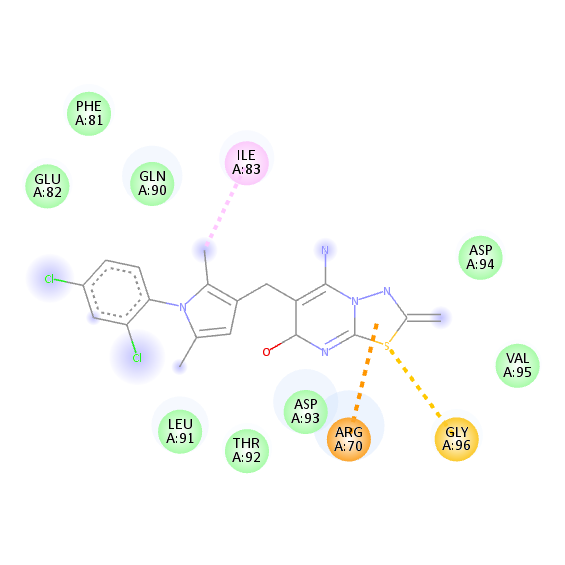 |
| ASA 30 ns | ASA 40 ns |
| 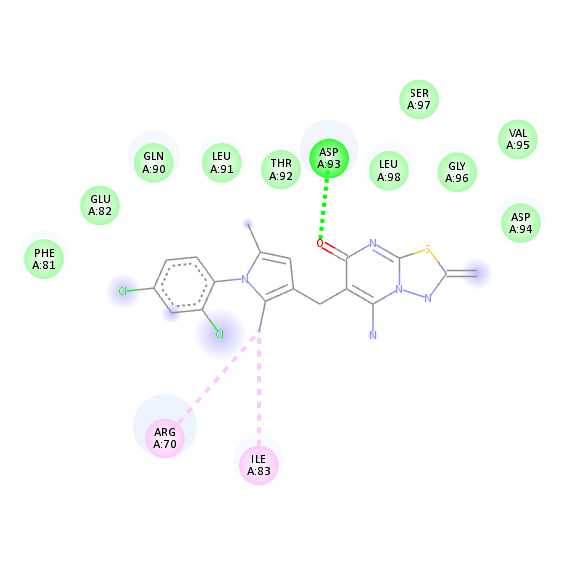 |  |
| ASA 50 ns |  |
| 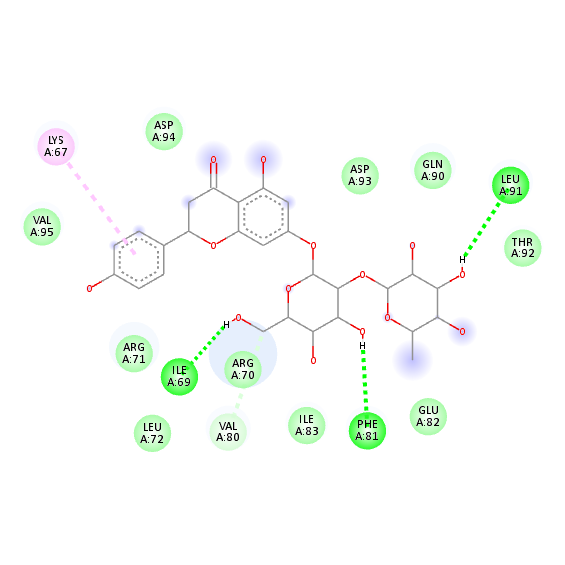 | 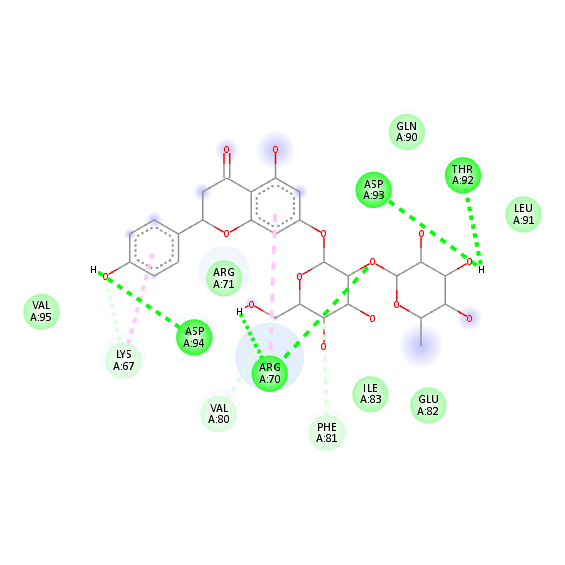 |
| NAR 10 ns | NAR 20 ns |
| 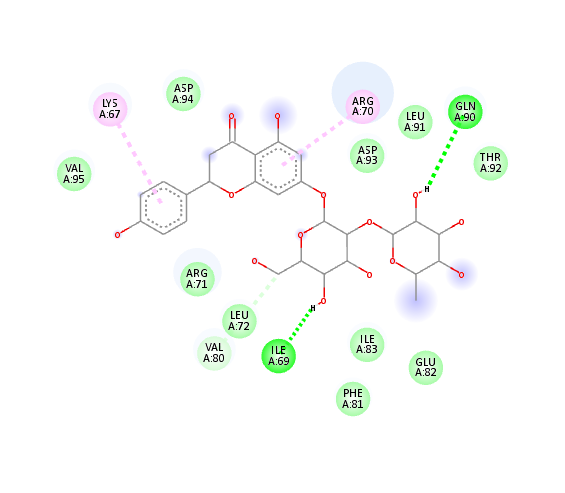 | 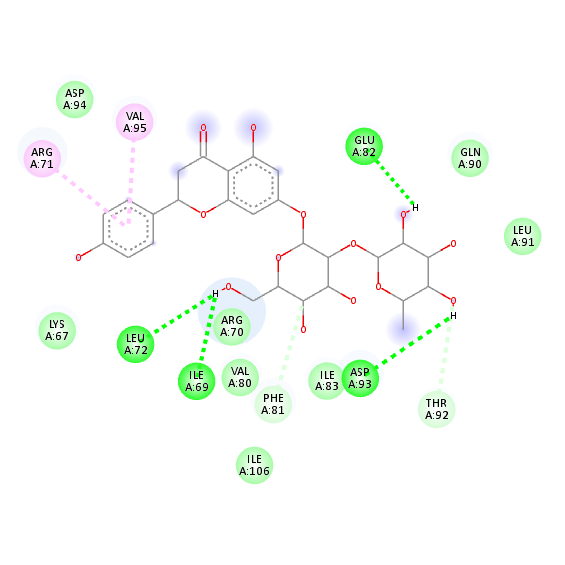 |
| NAR 30 ns | NAR 40 ns |
| 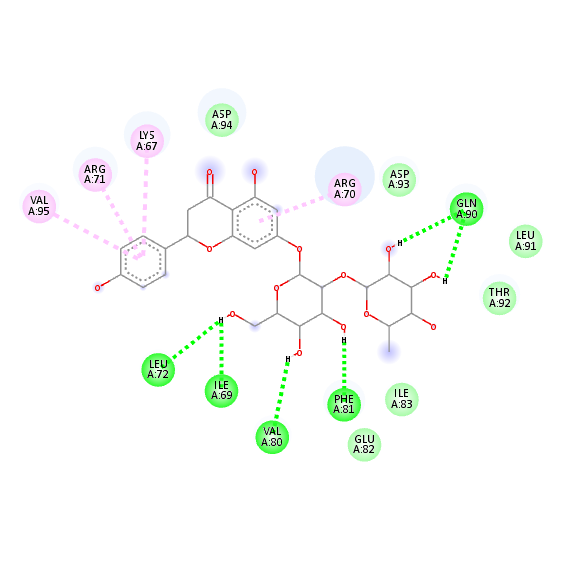 |  |
| NAR 50 ns |  |
| 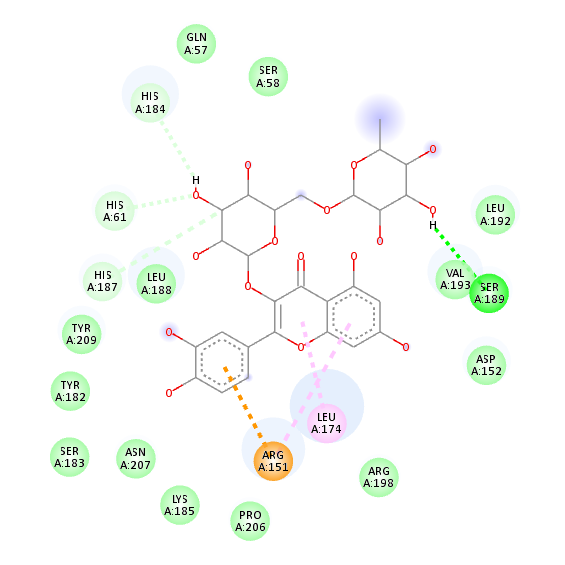 | 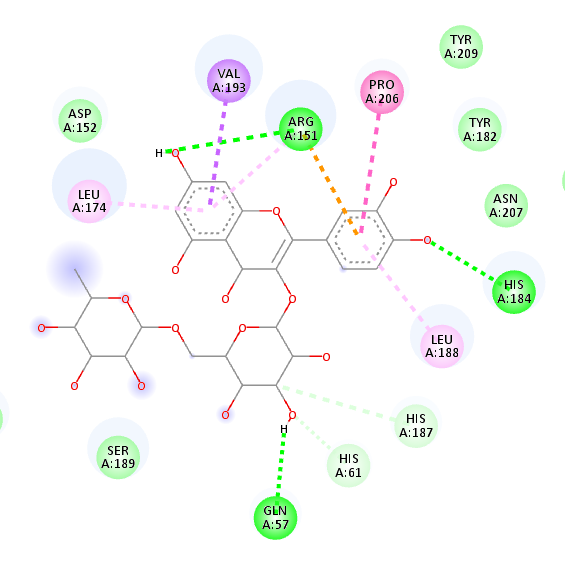 |
| RUT 10 ns | RUT 20 ns |
| 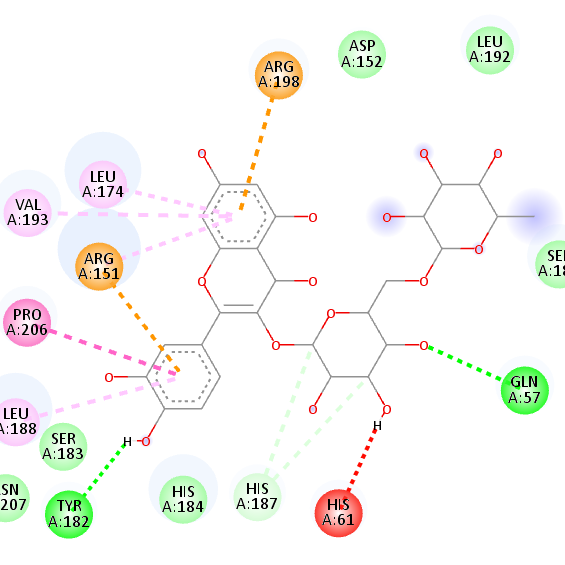 | 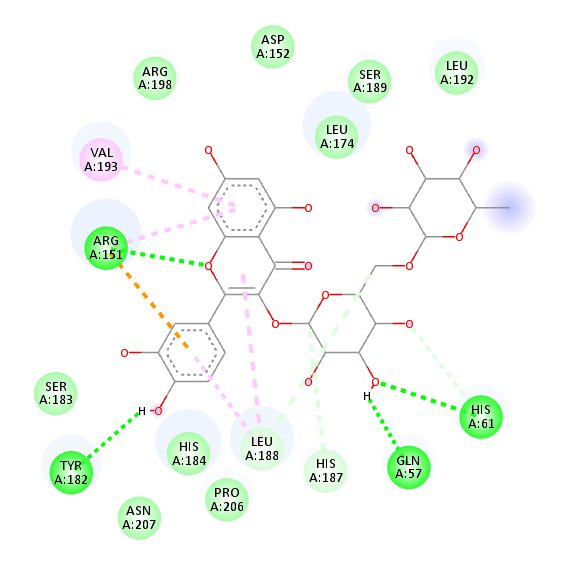 |
| RUT 30 ns | RUT 40 ns |
| 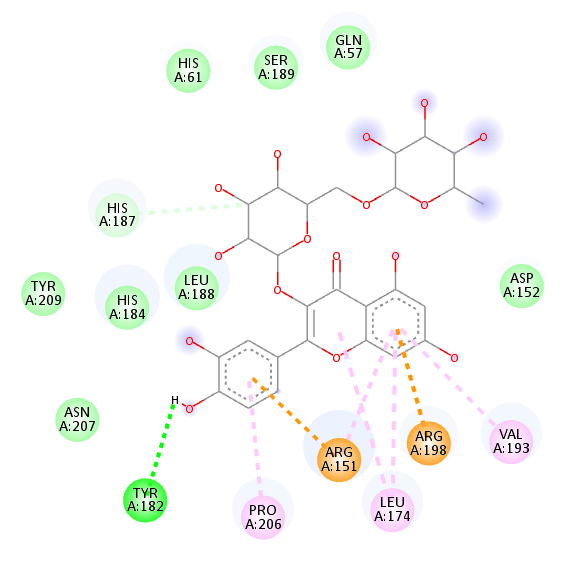 |  |
| RUT 50 ns |  |

| 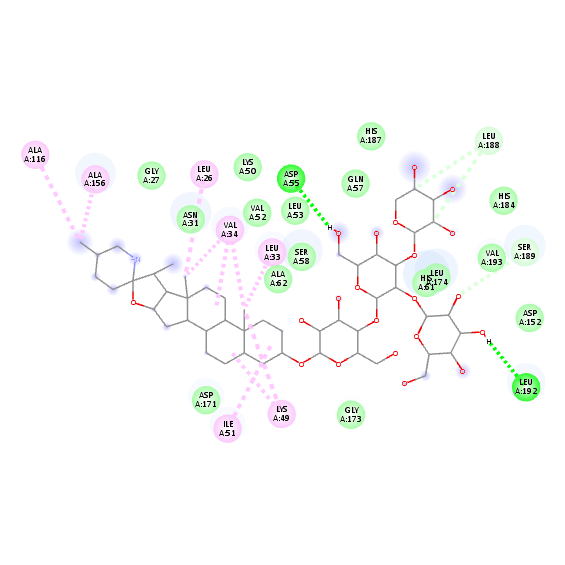 | 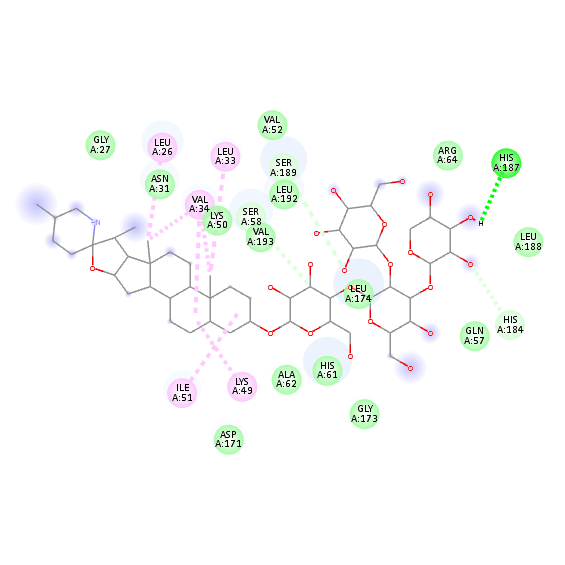 |
| --- | --- |
| TOM 10 ns | TOM 20 ns |
| 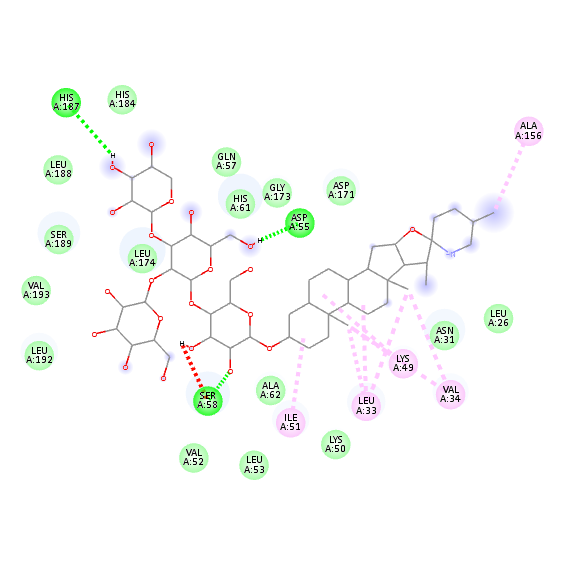 | 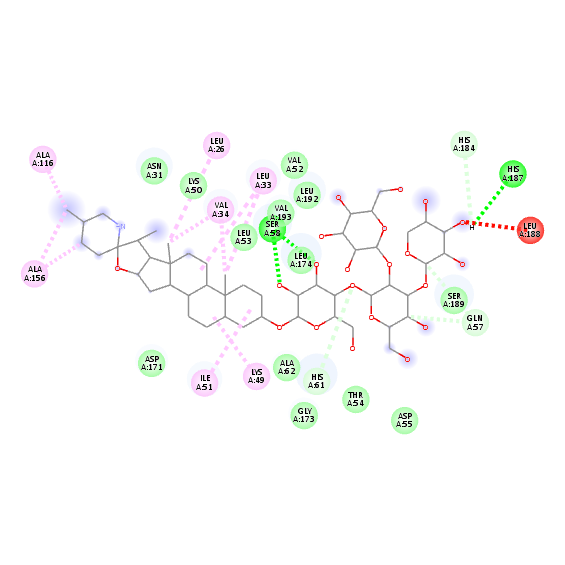 |
| TOM 30 ns | TOM 40 ns |
| 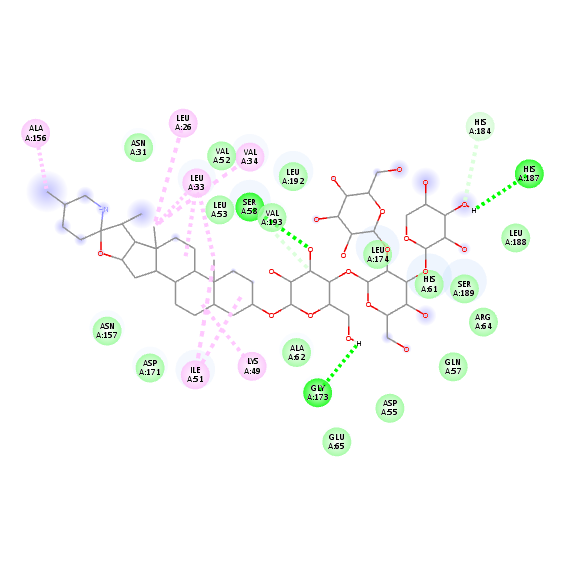 |  |
| TOM 50 ns |  |

| 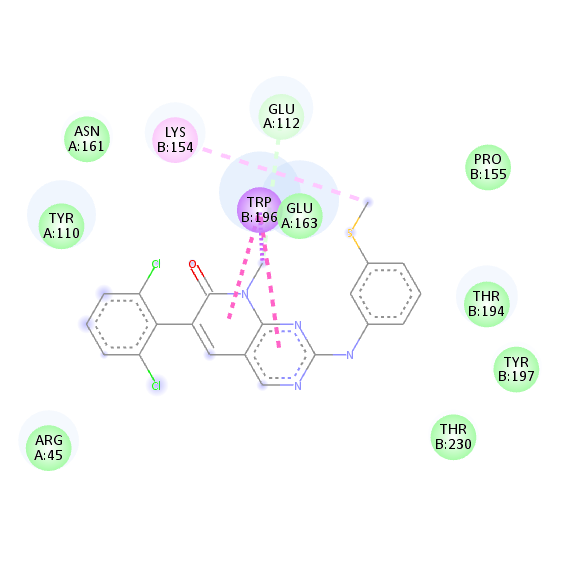 | 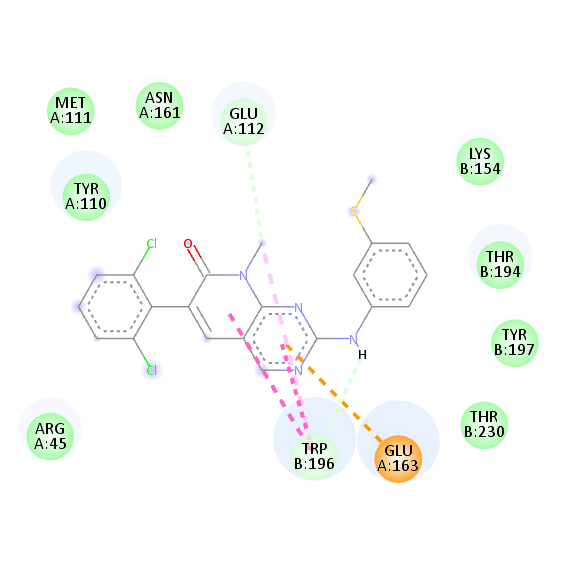 |
| --- | --- |
| STD 10 ns | STD 20 ns |
| 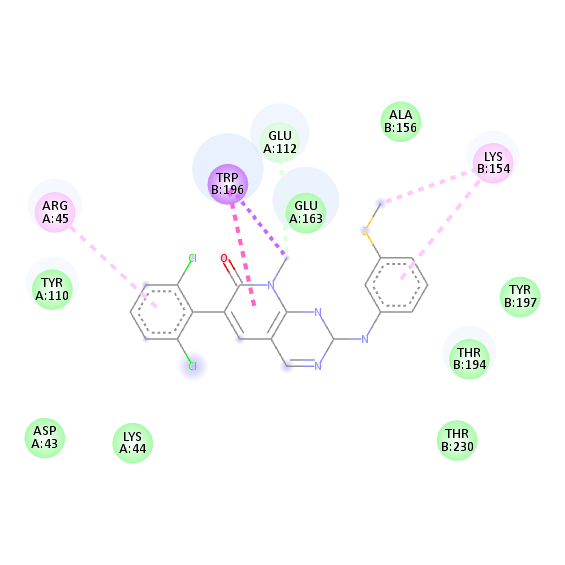 | 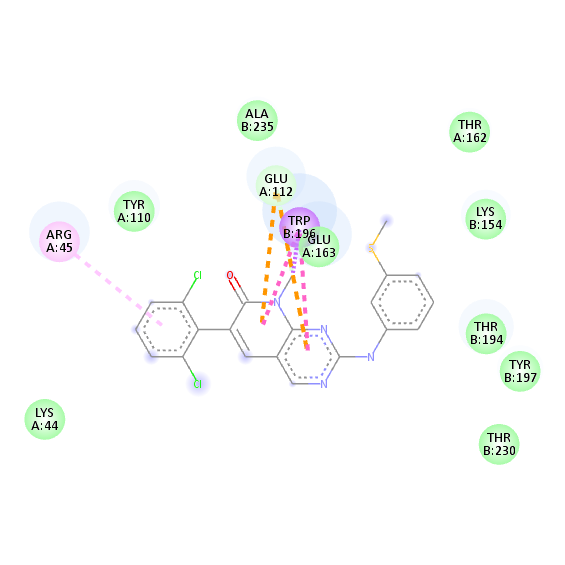 |
| STD 30 ns | STD 40 ns |
| 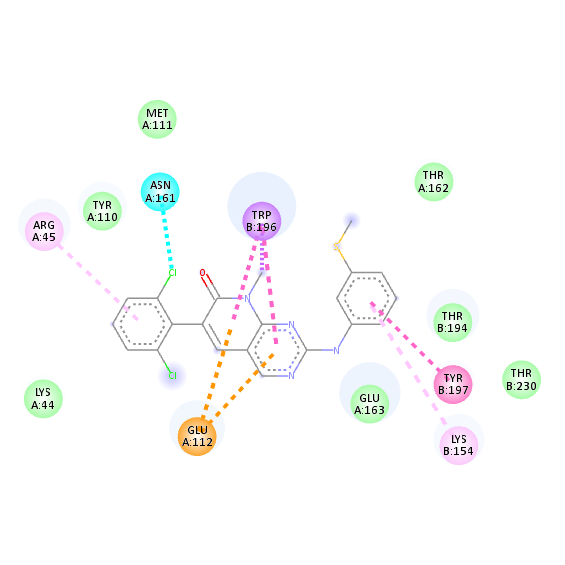 |  |
| STD 50 ns |  |
