# Supplementary material for: Pharmacoinformatics-based investigation of bioactive compounds of Rasam (South Indian recipe) against human cancer
Source: Sci Rep. 2021 Nov 2;11:21488. doi: 10.1038/s41598-021-01008-9 (PMC8563928; doi:10.1038/s41598-021-01008-9)
Supplement: Supplementary file 2 — Supplementary Information 2. [file 41598_2021_1008_MOESM2_ESM.doc]

Backup data

| S.No | 10NS | 20NS | 30NS | 40NS | 50NS |
| --- | --- | --- | --- | --- | --- |
| ASA | ARG70  ILE83  ASP93  GLY96 | ARG70  ILE83  ASP93  VAL95 | ARG70  ILE83  ASP93 | ARG70  ILE83  GLY96 | ARG70  ILE83  ASP93 |
| NAR | LYS67  ILE69  ARG70  PHE81  LEU91 | LYS67  ARG70  PHE81  THR92  ASP93 | LYS67  ILE69  ARG70  LEU72  GLN90 | ILE69  ARG70  ARG71  LEU72  PHE81  GLU82  THR92  VAL95 | LYS67  ILE69  ARG70  ARG71  LEU72  VAL80  PHE81  GLN90  VAL95 |
| RUT | HIS61  ARG151  LEU174  HIS184  HIS187  SER189 | GLN57  HIS61  ARG151  LEU174  HIS184  HIS187  LEU188  VAL193  PRO206 | GLN57  HIS61  ARG151  LEU174  HIS187  LEU188  VAL193  ARG198  PRO206 | GLN57  HIS61  ARG151  TYR182  HIS187  LEU188  VAL193 | ARG151  LEU174  TYR182  HIS187  LEU188  VAL193  ARG198  PRO206 |
| TOM | LEU26  LEU33 VAL34 LYS49  ILE51  ASP55 ALA116  ALA156  LEU188 LEU192  VAL193 | LEU26  LEU33 VAL34 LYS49  ILE51 HIS184  HIS187  LEU192  VAL193 | LEU33 VAL34 LYS49  ILE51  ASP55  SER58 ALA156  HIS187 | LEU26  LEU33 VAL34  GLN57  SER58  HIS61  ALA116  ALA156  HIS184  HIS187  SER189 | LEU26  LEU33 VAL34  LYS49  ILE51  SER58  ALA156  GLY173 HIS184  HIS187  VAL193 |
| STD | GLU112  LYS154  TRP196 | GLU112  GLU163  TRP196 | ARG45  GLU112  LYS154  TRP196 | ARG45  GLU112  GLU163  TRP196 | ARG45  GLU112  LYS154  ASN161  TRP196  TYR197 |
